# STARD12/14 are diagnostic and prognostic biomarkers of lung adenocarcinoma associated with epigenetic regulation, immune infiltration and ferroptosis

**DOI:** 10.7150/ijms.84566

**Published:** 2023-09-11

**Authors:** Wen-Di Zhang, Dong-Mei Hu, Zhuang-E Shi, Qing-Xiang Wang, Meng-Yu Zhang, Jian-Yu Liu, Xiu-Li Ji, Yi-Qing Qu

**Affiliations:** 1 Department of Pulmonary and Critical Care Medicine, Qilu Hospital, Cheeloo College of Medicine, Shandong University, Shandong Key Laboratory of infectious respiratory diseases, Jinan, China.; 2 Department of Pulmonary Disease, Jinan Traditional Chinese Medicine Hospital, Jinan, China.; 3 Department of Pulmonary and Critical Care Medicine, Qilu Hospital of Shandong University, Shandong Key Laboratory of infectious respiratory diseases, Jinan, China.

**Keywords:** lung adenocarcinoma (LUAD), prognosis, epigenetic regulation, m6A modification, ferroptosis, immune infiltration

## Abstract

**Background:** Metabolic reprogramming plays an important role in tumor progression and antitumor immunity. START domain-containing proteins (STARDs) are responsible for lipid metabolism. However, the underlying functions of STARDs in lung adenocarcinoma (LUAD) have not been clarified yet.

**Methods:** Oncomine, UALCAN, TCGA and CPTAC were used to explore the expression landscape and clinicopathological characteristics of STARDs in LUAD. Diagnostic and prognostic values were assessed by Kaplan-Meier Plotter, Cox regression analysis, and ROC curve. GeneMANIA, GO, KEGG and GSEA were applied for exploring the potential biological functions. Epigenetic process, including mutation and m6A modification were analyzed by cBioPortal and TCGA. TIMER, TISIDB and TCGA cohort provided an immune signature. The correlation between STARDs expression and ferroptosis was analyzed by TCGA. Finally, the STARDs expression were confirmed by RT-qPCR and western blot.

**Results:** STARD5/10/14 were overexpressed in LUAD compared with normal, while STARD4/7/8/11/12/13 were relatively low. STARD5/12/14 levels were positively related to clinical and lymph node stage. Survival analysis showed high STARD12 expression was associated with favorable overall survival, disease special survival as well as disease free survival, while STARD14 showed the opposite. GSEA analysis found STARD12 and STARD14 were associated with glycolysis, oxidative phosphorylation and tumor related signaling pathways. STARD12 co-expressed genes participated in cell cycle and DNA replication, and STARD14 were enriched in ECM-receptor interaction. Both STARD12 and STARD14 were corelated with epigenetic regulation, especially TP53 mutation and m6A modification. STARD12 expression was positively correlated with TMB level. The level of STARD12 was significantly associated with the abundance of infiltrating immune cells, including B cells, CD8+T cells, macrophages, dendritic cells, and chemokine, receptor, MHC, immunostimulatory related genes. STARD14 was negatively associated with the infiltration of CD8+T cells, while positively with CCL28 and immune checkpoints, including CTLA4 as well as PD-L2. In addition, STARD12/14 could regulate the ferroptosis related genes.

**Conclusion:** STARD12 and STARD14 were expected to be potential biomarkers for LUAD, which were associated with epigenetic regulation, immune infiltration and ferroptosis.

## Introduction

Lung cancer is the first leading diagnosed malignant tumor globally 1. Non-small cell lung cancer (NSCLC) accounts for 85-90% of cases, while lung adenocarcinoma (LUAD) is the most common subtype 2. Although advanced treatments including targeted therapy and immunotherapy have developed, such as Crizotinib, Osimertinib, Pembrolizumab, and Atezolizumab, the 5-year overall survival rate of patients with NSCLC is still less than 20% 2. Therefore, more accurate prognosis biomarkers or therapy targets remain the primary focus in LUAD research.

Tumor metabolic reprogramming is considered an emerging hallmark of cancer. Tumor cells have essential requirements for cellular building blocks, including nucleic acids, lipids, and proteins. To adapt to the unlimited proliferation, tumor cells often undergo epigenetic regulation and metabolic alterations: not only confined to the Warburg effect centered on glycolysis and the tricarboxylic acid cycle, but also involving lipid and glutamate metabolism 3. These metabolic alterations result in an acidic and hypoxic tumor microenvironment, which compete and limit the energy and nutrient to immune infiltrating cells, and in turn hamper the anti-tumor immune response and participate in tumor progression 4. The START domain-containing protein (STARD) family encoding genes function as important lipid transfer proteins (LTPs) that play an important role in regulating lipid metabolism. The START domain is a conserved sequence of 210 residues with an α/β helix-grip structure 5. STARDs contain 15 proteins in Homo sapiens and are categorized into six subfamilies via sequence homology 6. STARD4/5/6 can bind cholesterol or oxysterols, and STARD2/7/10/11 are able to combine phospholipids or sphingolipids. STARD9 owns kinetin motor function; STARD8/12/13, also known as DLC protein family, shares the Rho-GTPase domain and STARD14/15 are newly discovered with thioesterase activity. Existing researches have proved STARDs are critical regulators of tumor proliferation, and metastasis 6. However, the underlying functions of STARDs in patients with LUAD were little explored.

**Figure 1:**
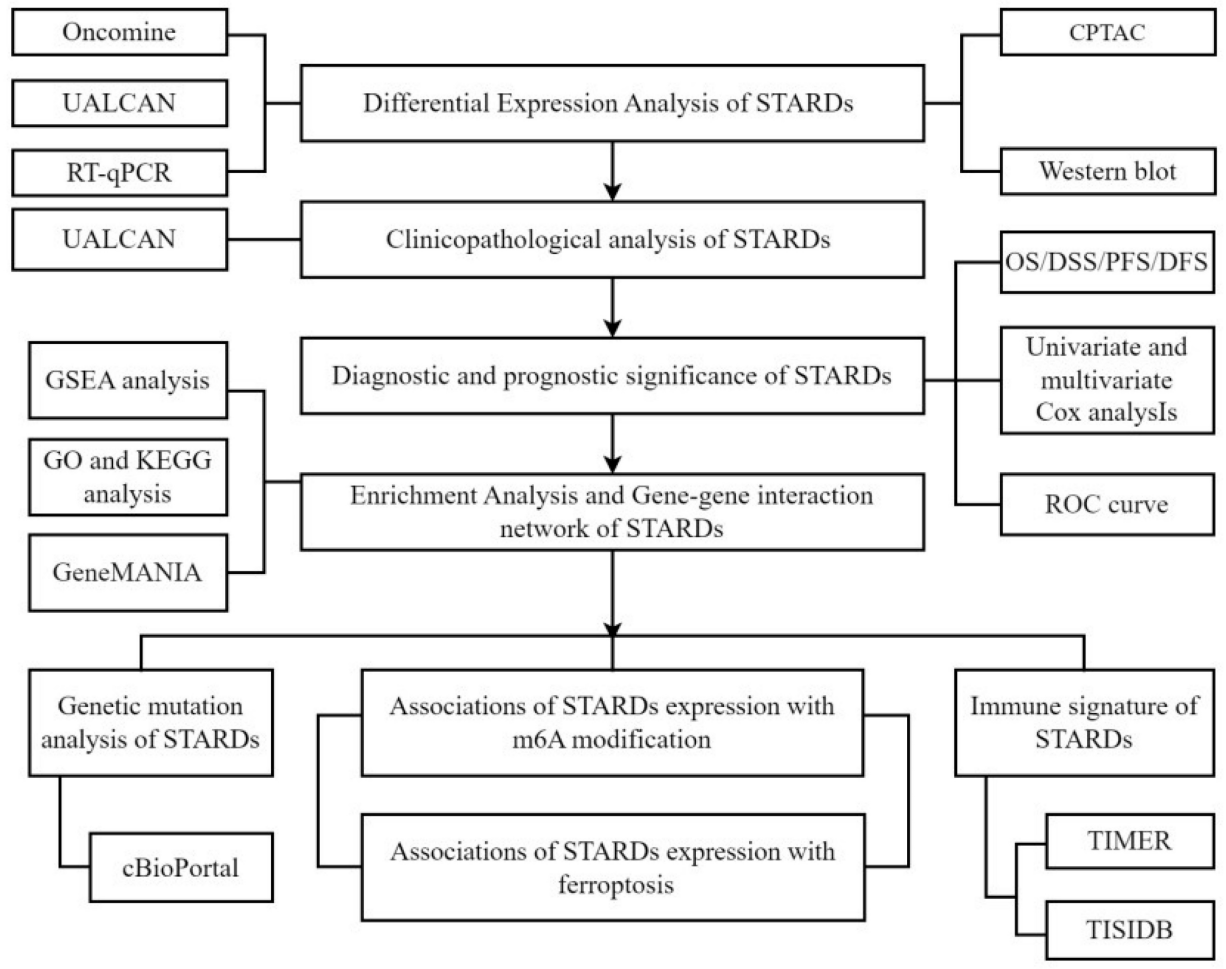
**Analytic flowchart.** The study mainly comprised eight parts: I Differential expression analysis of STARDs in LUAD; II Clinicopathological analysis of STARDs in LUAD; III Diagnostic and prognostic significance of STARDs expression in LUAD; IV Enrichment Analysis and Gene-gene interaction network of STARDs in LUAD; V Genetic mutation analysis of STARDs in LUAD; VI Association of STARDs expression with m6A modification in LUAD; VII Immune signature of STARDs in LUAD; VIII Association of STARDs expression with ferroptosis in LUAD. GSEA, Gene Set Enrichment Analysis; GO, Gene ontology; KEGG, Kyoto Encyclopedia of Genes and Genomes.

Hence, we determined to provide a comprehensive assessment of STARDs in LUAD, including prognostic value, epigenetic as well as immune infiltration characteristics. In addition, studies have found that ferroptosis is closely related to lipid metabolism, and the metabolites produced by ferroptosis could have multiple molecular crosstalk with immune cells and molecules in the tumor immune microenvironment 7. The association with ferroptosis was also analyzed in our study. Our analytic workflow was briefly outlined in Figure 1.

## Materials and methods

### Material datasets

The datasets were obtained from The Cancer Genome Atlas (TCGA), which is a coordinated project offering sequencing and pathological data of common human cancers to improve diagnosis and treatment to prevent cancer. The datasets for analysis were as followed: The Cancer Genome Atlas Lung Adenocarcinoma (TCGA-LUAD) dataset including RNA-seq expression data, simple nucleotide variation (SNV) data (https://portal.gdc.cancer.gov/), phenotypic and survival data (https://xenabrowser.net). Besides, we used CPTAC dataset (https://cptac-data-portal.georgetown.edu/datasets), a national platform on cancer via the application of proteogenomics, to gather expression information on protein level.

### Differential expression and clinicopathological analysis

According to the ONCOMINE (https://www.oncomine.org/) dataset, we tested the transcription levels of STARDs in 20 different types of cancer diseases 8. UALCAN (http://ualcan.path.uab.edu.) is another interactive web-portal to perform to in-depth analyses based on level 3 RNA-seq and clinical data of 31 cancer types from TCGA database 9, allowing us to explore the differential expressions of STARDs in LUAD between tumor and normal samples. In addition, the relationships between mRNA expression of STARDs with clinicopathological parameters of LUAD patients were also analyzed. Further we conducted differential expression analysis of the STARDs family in LUAD according to histological classification.

### Survival analysis

We used Kaplan Meier plotter (http://kmplot.com/analysis/), a network aimed to discover and validate survival biomarkers meta-analysis-based 10, to assess the effect of mRNA expression of STARDs on the prognosis of LUAD patients. Additionally, we obtained the time nodes of disease progression and recurrence in the TCGA-LUAD cohort, and conducted correlation analysis between the expression levels of STARDs, DFS (Disease Free Survival) and PFS (Progress Free Survival) in LUAD patients. The survival analysis on DSS (Disease Special Survival) was also explored.

### Enrichment and interaction analysis

First, we used Gene ontology (GO) and Kyoto Encyclopedia of Genes and Genomes (KEGG) analyses to identify the potential function of STARDs and similar genes. Similar genes were gained from GEPIA. GO is the standardized representation of gene and gene products, covering three domains: Cellular components, molecular function, and biological process 11 and the KEGG is the knowledge base for gene functions systemic analysis 12. The GeneMANIA (http://genemania.org/) prediction server offers biological network integration for STARDs 13. Further, we carried out Gene Set Enrichment Analysis (GSEA) (www.gsea-msigdb.org/gsea/index.jsp) (Subramanian et al., 2005) to evaluate the related pathways and molecular mechanisms of STARD12/14 in LUAD. GSEA analysis was performed using GSEA software (version 3.0). We grouped samples by median and selected h.all.v7.4.syndromes.gmt as reference gene set. GO and KEGG analysis of STARD12/14 co-expression genes was used by R-clusterProfiler package. The co-expressed genes (|cor| > 0.35 and p value < 0.05) were collected from cBioportal. p value of < 0.05 and FDR of < 0.25 were considered statistically significant. Results were visualized using the R-ggplot2 package.

### Mutational landscape of STARDs

The cBioPortal (http://cbioportal.org) provides a resource for exploring, visualizing, and analyzing multidimensional cancer genomics data 14, 15. In this study, cBioPortal was applied to investigate genetic alterations and clinical attributes of STARDs. Waterfall plot described the mutational landscape of the top 15 genes with the most frequent mutations in the high and low expression groups of STARD12/14 in LUAD. TMB (Tumor mutation burden) of LUAD samples was calculated using the tmb function of R-maftools R package. The relationship between gene expression and mutations and TMB of STARD12/14 was analyzed by R-maftools package.

### Association of STARDs expression with m6A modification

20 m6A modification related genes, including YTHDF1, WTAP, RBM15, FTO, ALKBH5, ZC3H13, YTHDF3, HNRNPC, YTHDC2, METTL14, METTL3, IGF2BP3, IGF2BP2, RBMX, RBM15B, IGF2BP1, YTHDC1, HNRNPA2B1, VIRMA, and YTHDF2 16 were incorporated into our study. First, Spearman correlation coefficient was calculated to explore the correlation between STARD12, STARD14 and m6A related gene mRNA expression levels. The differential expression of m6A related genes in low and high STARD12/14 expression group were tested by Wilcoxon-Mann-Whitney test. The Logrank regression detected the relationship of expression levels of m6A related genes with the OS of LUAD patients. *p* < 0.05 was considered statistically significant. Results were visualized using the R-ggplot2 package.

### Immune signature of STARDs

The relationship between STARD12/14 expression levels and tumor purity, stromal score and immune score was used via R-estimate package. TIMER (https://cistrome.shinyapps.io/timer/) is designed to analyze systematically of immune infiltrates across diverse cancers 17, which allowed us to explore the association between STARD12/14 expression levels and the abundance of Immune infiltrating cells in LUAD. The somatic copy number alteration module was carried to explore the relationships between the abundance of immune infiltrates and somatic copy number variation (CNV). We used TISIDB (http://cis.hku.hk/TISIDB/), another integrated portal for tumor-immune system interactions 18, to explore different immune subtype of STARD12 and STARD14 in LUAD. We further calculated the association between STARD12/14 expression level and 150 immune-related genes, which includes five categories, that is, chemokine, receptor, MHC, immunoinhibitory and immunostimulatory.

### Association of STARDs expression with ferroptosis

We selected 25 ferroptosis related genes based on the literature, including CDKN1A, HSPA5, EMC2, SLC7A11, NFE2L2, MT1G, HSPB1, GPX4, FANCD2, CISD1, FDFT1, SLC1A5, SAT1, TFRC, RPL8, NCOA4, LPCAT3, GLS2, DPP4, CS, CARS, ATP5MC3, ALOX15, ACSL4 and AIFM2^19^. First, Spearman correlation coefficient was calculated to explore the correlation between STARD12, STARD14 and ferroptosis related genes expression levels. We then divided TCGA-LUAD samples into high and low expression group according to the expression level of STARD12/STARD14. The differential expression of ferroptosis related genes was tested by Wilcoxon-Mann-Whitney test. The Logrank regression detected the relationship of expression levels of ferroptosis related genes with the OS of LUAD patients. p < 0.05 was considered statistically significant. Results were visualized using the R-ggplot2 package.

### Cell culture and Quantitative real-time PCR

Human lung epithelial cell line (BEAS-2B Cell Article: No.CL-0496) and LUAD cell lines (NCI-H1299 Cell Article: No.CL-0165, A549 Cell Article: No.CL-0016 and PC9 Cell Article: No.CL-0298) were purchased from Procell Life Science & Technology Co. Ltd. (Wuhan, China) on December 10, 2021. The BEAS-2B and PC9 were cultured in Dulbecco's modified Eagle's medium (DMEM, Gibco). A549 and NCI-H1299 were placed in RPMI 1640 (Gibco). The culture mediums were both supplemented with 10% fetal bovine serum (FBS) and the cells were cultured at 37°C containing 5% CO2. Total RNA was extracted from cell lines via TRIzol reagent (Invitrogen). Evo M-MLV RT Master Mix (Takara) was applied to reverse-transcribed mRNA into cDNA. Quantitative real-time PCR (RT-qPCR) was performed with SYBR Premix Ex Taq II (Takara) on Bio-Rad. The primer sequences of the GAPDH, STARD12 and STARD14 were listed in Supplementary Table 1.

### Protein extraction and Western blot

The total protein was extracted by radioimmunoassay (RIPA) buffer (Beyotime, China) and Phenylmethanesulfonyl fluoride (PMSF) Solution (Beyotime, China). 10% SDS-PAGE was used for protein separation. Then transferred to PVDF membranes and sealed with 5% skimmed milk powder. The primary antibody was incubated in a 4 °C shaking table, then incubated with HRP coupled secondary antibody, and finally developed with ECL kit (Millipore, German). The antibodies included STARD12 (66894-1-lg, Proteintech), STARD14 (ab180745, abcam), GAPDH (10494-1-AP, Proteintech) and HRP-labeled Goat anti-mouse and anti-rabbit IgG (zsbio, China).

### Statistical analysis

The statistical data were analyzed using R, SPSS Statistics 25.0 and GraphPad Prism 9.3.1. The differential expression in different subgroups was analyzed by the Wilcoxon rank-sum test. The survival analysis was performed using Kaplan-Meier survival, univariate and multivariate Cox regression analysis. The independent variables p < 0.2 were retained for multivariate Cox regression analysis. Spearman or Pearson correlation coefficient was calculated to explore the correlation with m6A, ferroptosis related genes and immune signatures. The comparative CT method (2-ΔΔCT) was used to calculate the relative expression levels of the qRT-PCR data. Ordinary one-way ANOVA was used to perform differential analysis. p < 0.05 was considered statistically significant.

## Results

### Differential expression analysis of STARDs in LUAD

**Figure 2:**
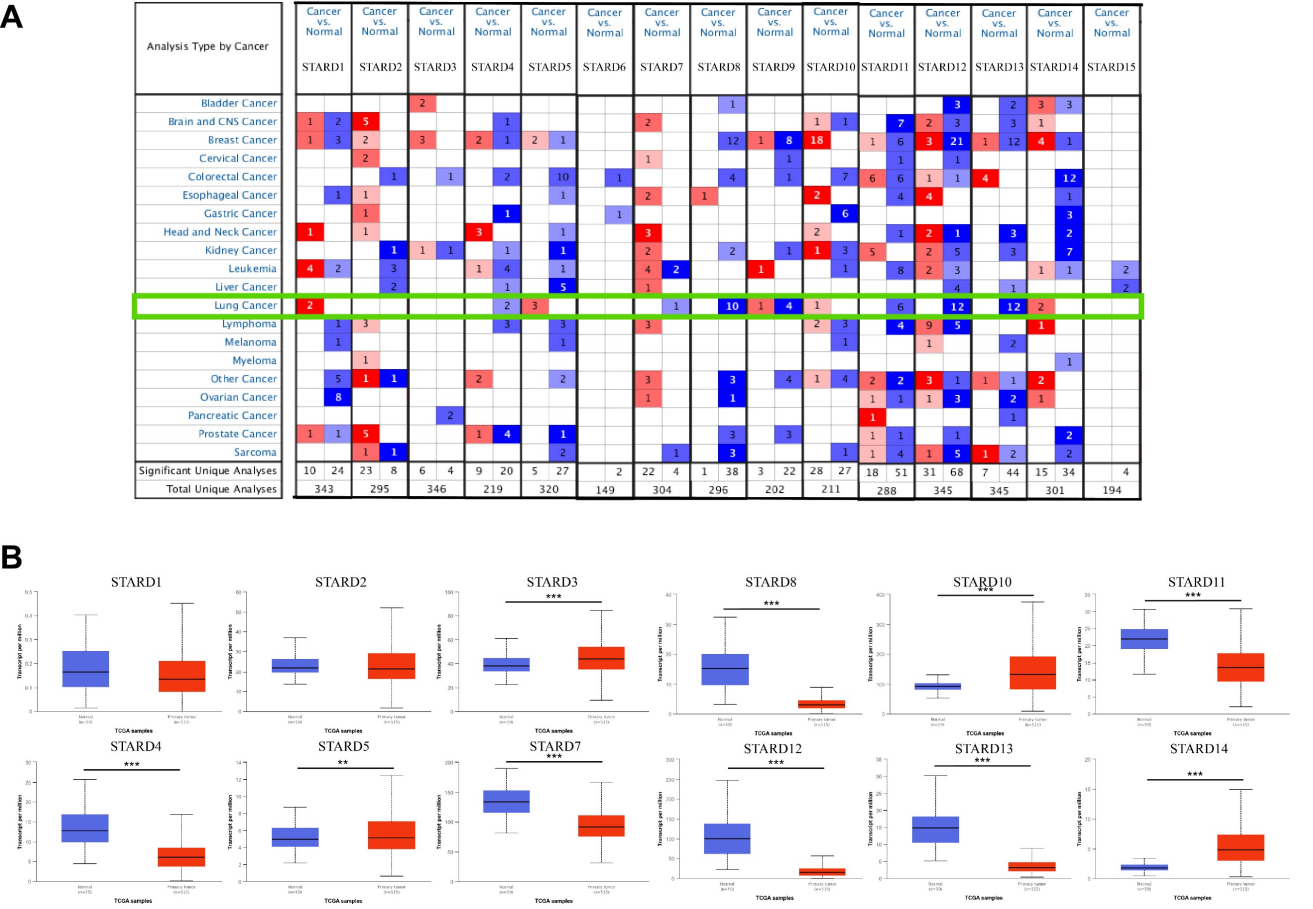
** Transcriptional and clinical landscape of STARDs in LUAD**. (**A**) The mRNA expression of STARDs in 20 cancer types (Oncomine). The cut-off of p value and fold change were as following: p value: 0.01, fold change: 1.5, gene rank: 10%, data type: mRNA. (**B**) Expression of STARDs in LUAD (UALCAN). The transcription levels of STARD3/5/10/14 in LUAD samples were higher than normal samples, whereas STARD4/7/8/11/12/13 were lower.

To explore the differential expression of STARDs between LUAD and normal samples, we performed analyses via Oncomine and UALCAN. As shown in Figure 2A and Supplementary Table 2, the mRNA expressions of STARDs in 20 types of cancers compared with normal samples were assessed. In LUAD, significantly higher mRNA expressions of STARD1/5/9/10/14 were found in multiple datasets. In Garber Lung dataset, remarkable overexpression of STARD1 was found in LUAD tissues compared to normal with a fold change of 3.779 (p=7.15E-7). Up-expression of STARD5 was observed in Su Lung database with a fold change of 1.547 (p=3.80E-4). Similarly, the expression of STARD9 and STARD10 were 1.843-fold (p=7.62E-6) and 1.569-fold increased (p=8.62E-10), respectively. Su also found 2.069-fold increase of STARD14 mRNA expression in tumor tissues compared with normal tissues (p=9.99E-5), whereas the result from Selamat dataset showed 2.069-fold (p=4.91E-14). We further analyzed STARDs special expression characteristics in TCGA-LUAD cohort. Figure 2B illustrated higher transcription levels of STARD3/5/10/14 and lower expressions of STARD4/7/8/11/12/13 in LUAD samples compared with normal samples (all p<0.05). The results of pathological histological subgroup analysis were basically consistent with the overall analysis (Supplementary Figure 1).

### Clinicopathological analysis of STARDs in LUAD

**Figure 3:**
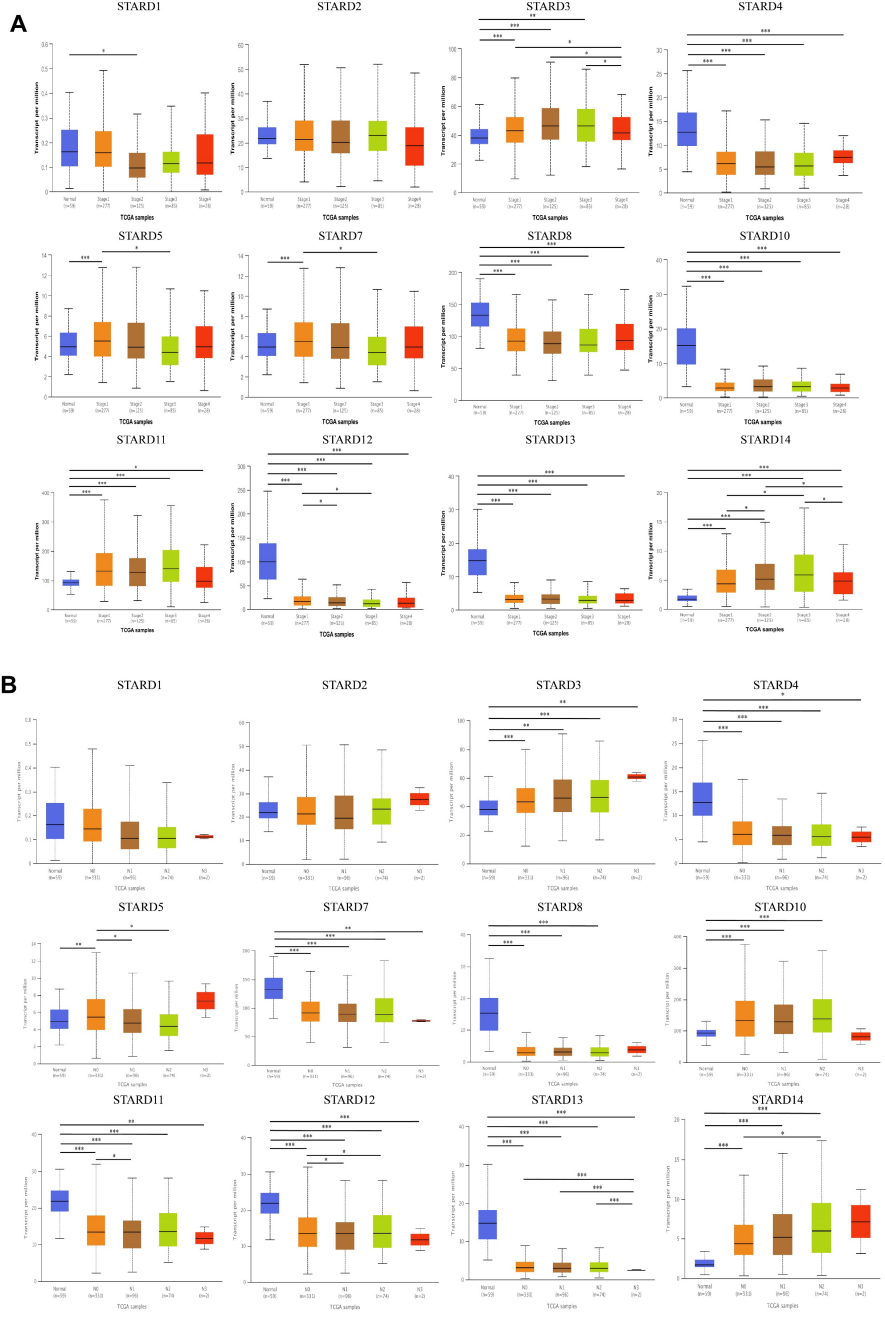
** Correlation between STARDs expression and clinicopathological parameters.** (**A**) The expression of STARDs differences between the clinical stages of LUAD and normal tissues. **(B)** The expression of STARDs differences between the lymph node stages of LUAD and normal tissues. *p<0.05, **p<0.01, ***p<0.001.

Then we analyzed the relationship between the differentially expressed STARDs with clinicopathological parameters of LUAD patients via UALCAN. Emphasizing on the available data, as were shown in Figure 3A, the mRNA expressions of STARD12 and STARD14 were prominently correlated with patients' individual cancer stages. Patients who were in more advanced cancer stages tended to have higher mRNA expression of STARD14 and lower STARD12. Similarly, the analysis of lymph node metastasis was done. As was shown in Figure 3B, the mRNA expression of STARD5 and STARD14 were significantly positively correlated with lymph node stages, but the peak of STARD14 was at stage N2, which might due to the small sample size (only 2 at stage N3). In contrast, STARD12 harbored a negative relationship.

### Diagnostic and prognostic significance of STARDs expression in LUAD

**Figure 4:**
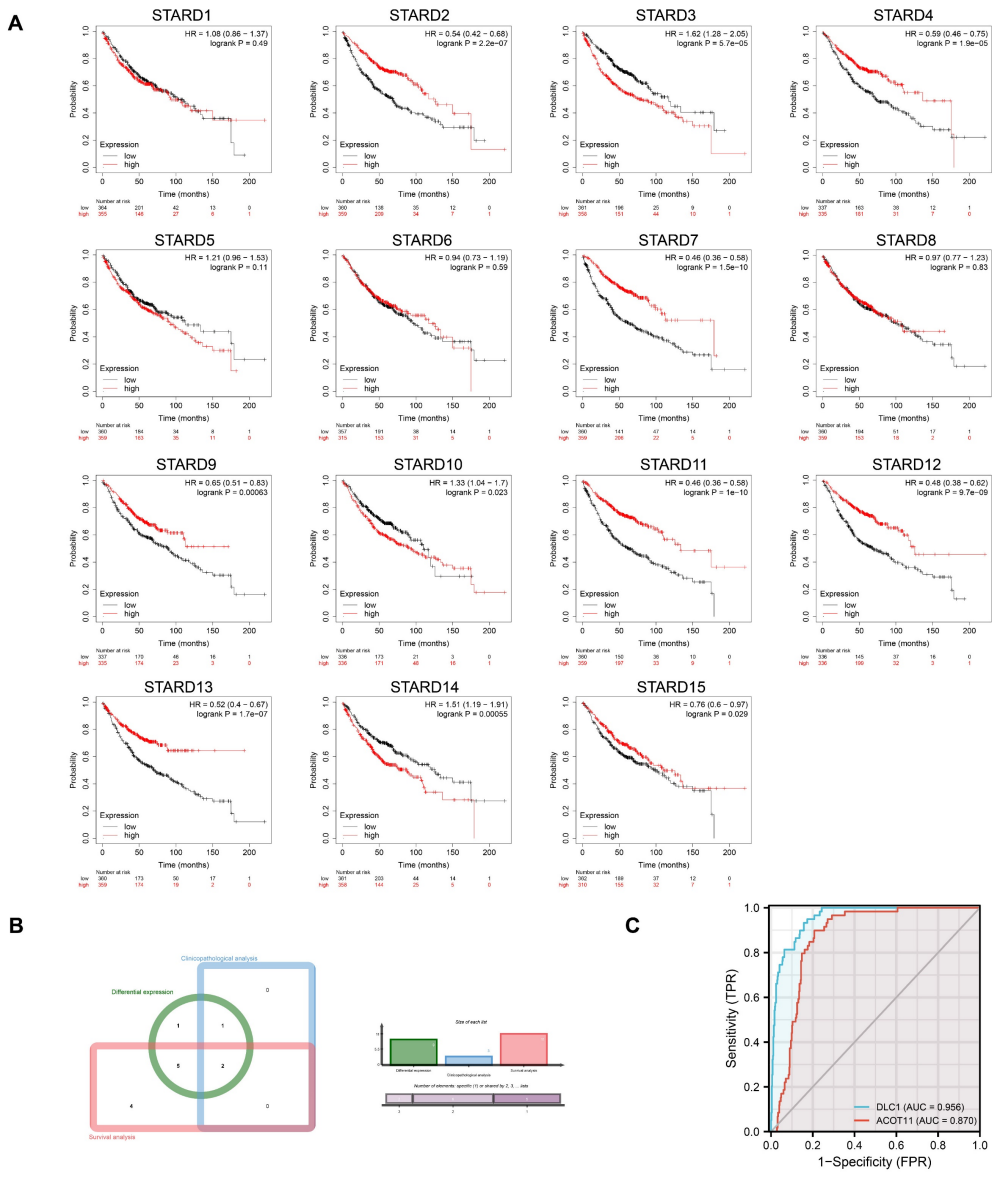
**Diagnosis and prognosis assessment of STARDs in LUAD.** (**A**) Overall survival differences based on mRNA expression (Kaplan-Meier plotter). STARD2/3/4/7/9/10/11/12/13/14/15 performed prognostic values. *p*<0.05. (**B**) Integrated analysis among transcriptional, clinical and survival information. (**C**) ROC curve showed STARD12/14 had good accuracy in predicting LUAD.

Kaplan-Meier plotter was adopted to assess the prognostic values of STARDs in LUAD patients. Figure 4A indicated that higher mRNA expression of STARD3 (HR=1.62, 95%CI:1.28-2.05, p=5.7e-05), STARD10 (HR=1.33, 95%CI:1.04-1.7, p=0.023), STARD14 (HR=1.51, 95%CI:1.19-1.91, p=0.00055) were significantly associated with worse OS of LUAD patients, while higher mRNA expression of STARD2 (HR=0.54, 95%CI:0.42-0.68, p=2.2e-07), STARD4 (HR=0.59, 95%CI:0.46-0.75, p=1.9e-05), STARD7 (HR=0.46, 95%CI:0.36-0.58, p=1.5e-10), STARD9 (HR=0.65, 95%CI:0.52-0.83, p=0.00063), STARD11 (HR=0.46, 95%CI:0.36-0.58, p=1e-10), STARD12 (HR=0.48, 95%CI:0.38-0.62, p=9.7e-09), STARD13 (HR=0.52, 95%CI:0.4-0.67, p=1.7e-07), STARD15 (HR=0.76, 95%CI:0.6-0.97, p=0.029) were significantly related to favorable OS of LUAD patients. However, mRNA expression of STARD1/5/6/8 showed no correlation with prognosis. Supplementary Figure 2 indicated that higher mRNA expression of STARD1 and STARD12 was significantly associated with favorable DFS of LUAD patients, while higher level of STARD14 was related to worse DFS though without obvious statistical difference (p=0.070). Higher mRNA expression of STARD1 and STARD5 were significantly associated with better PFS, higher level of STARD14 was significantly associated with worse PFS in LUAD patients (Supplementary Figure 3). Besides, as shown at Supplementary Figure 4, higher mRNA expression of STARD1/5/9/12 were significantly associated with favorable DSS in LUAD, whereas patients with higher level of STARD14 had worse DSS. Combined the comprehensive differential expression analysis, clinicopathological analysis and survival analysis, we suggested STARD12 and STARD14 as the most potential biomarker of STARDs in LUAD (Figure 4B). We evaluated the value of STARD12 and STARD14 in distinguishing normal from LUAD using ROC curves. The results showed that both STARD12 and STARD14 had good accuracy in predicting the incidence of LUAD (AUC = 0.953, CI = 0.934-0.973, AUC = 0.980, CI = 0.969-0.991, respectively) (Figure 4C). We also underwent univariate and multivariate Cox analysis to investigate whether STARD12 and STARD14, along with variables such as age, gender, smoke status and clinical stage classification, were risk factors for OS in LUAD patients (Supplementary Table 3). We found independent prognostic value of STARD12 expression (HR=0.846, 95%CI: 0.739-0.969, p= 0.015) in multivariate analysis, while STARD14 played limited role (HR= 1.169, 95%CI: 0.872-1.568, p=0.297).

### Enrichment Analysis and Gene-gene interaction network of STARDs

**Figure 5:**
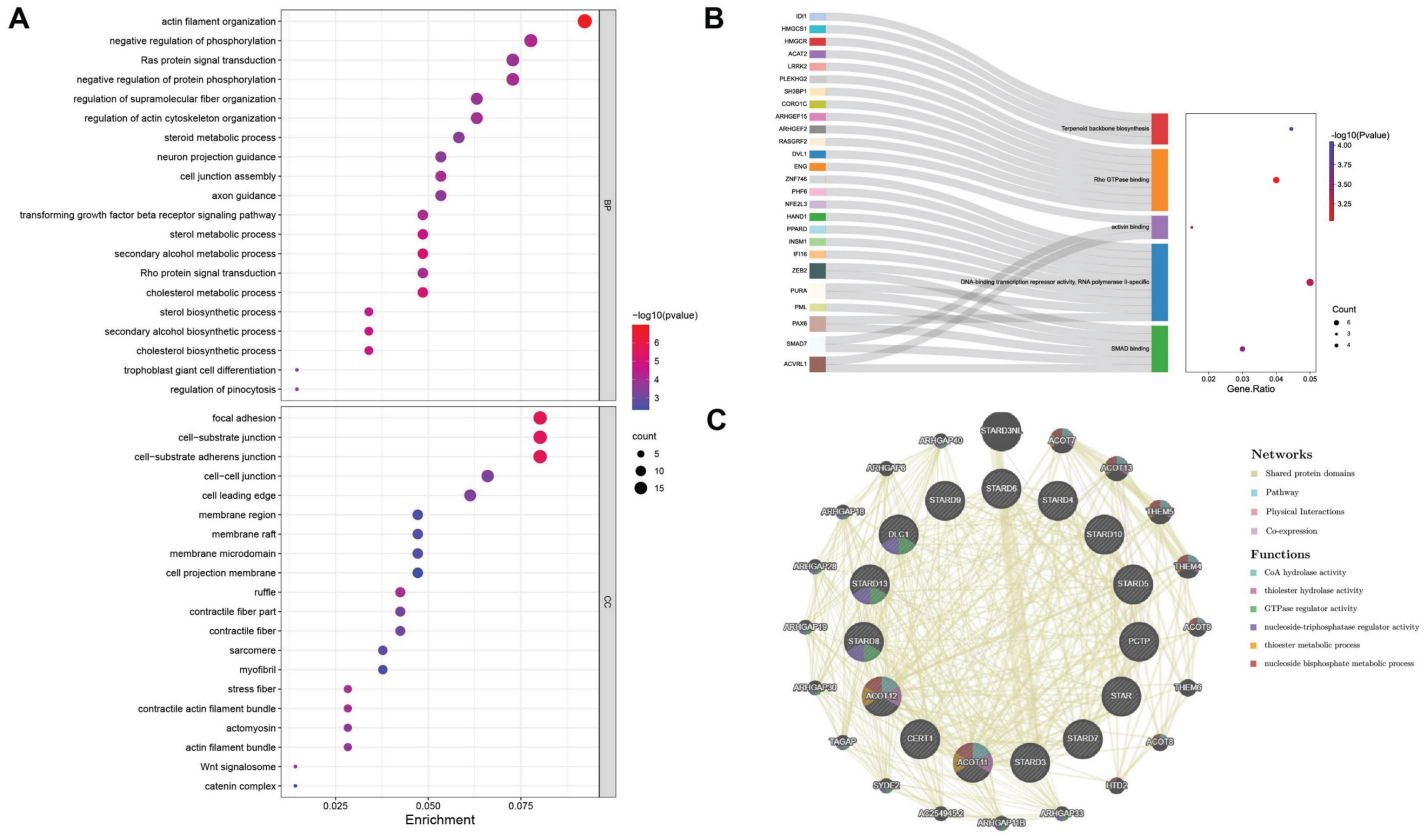
** Enrichment and interaction analysis of STARDs in LUAD**. (**A**) Bubble dot plot of cellular component and molecular function of STARDs and their neighboring genes via GO analysis. (**B**) Sankey dot of pathway enrichment of biological process via GO and KEGG analysis. (**C**) Gene-gene interaction network of STARDs (GeneMANIA).

The bubble chart and Sankey plot showed the top 20 cell components, molecular functions and top 5 biological processes (Figure 5A-5B). The GO analysis indicated that STARDs' similar genes were not only acted in lipid metabolism extensively, such as involved in cholesterol transfer and steroid biosynthetic process, they were also involved in TGF-β signaling pathway, cell junction, Rho and Ras protein signal transduction and transcription activity. In KEGG analysis, STARDs were enriched in Terpenoid backbone biosynthesis. There were 20 nodes surrounding 15 central nodes in the gene-gene interaction network, which revealed remarkable correlation with CoA hydrolase activity (FDR = 6.81e-12) and ARHGAP family genes, such as ARHGAP18 (Figure 5C).

**Figure 6:**
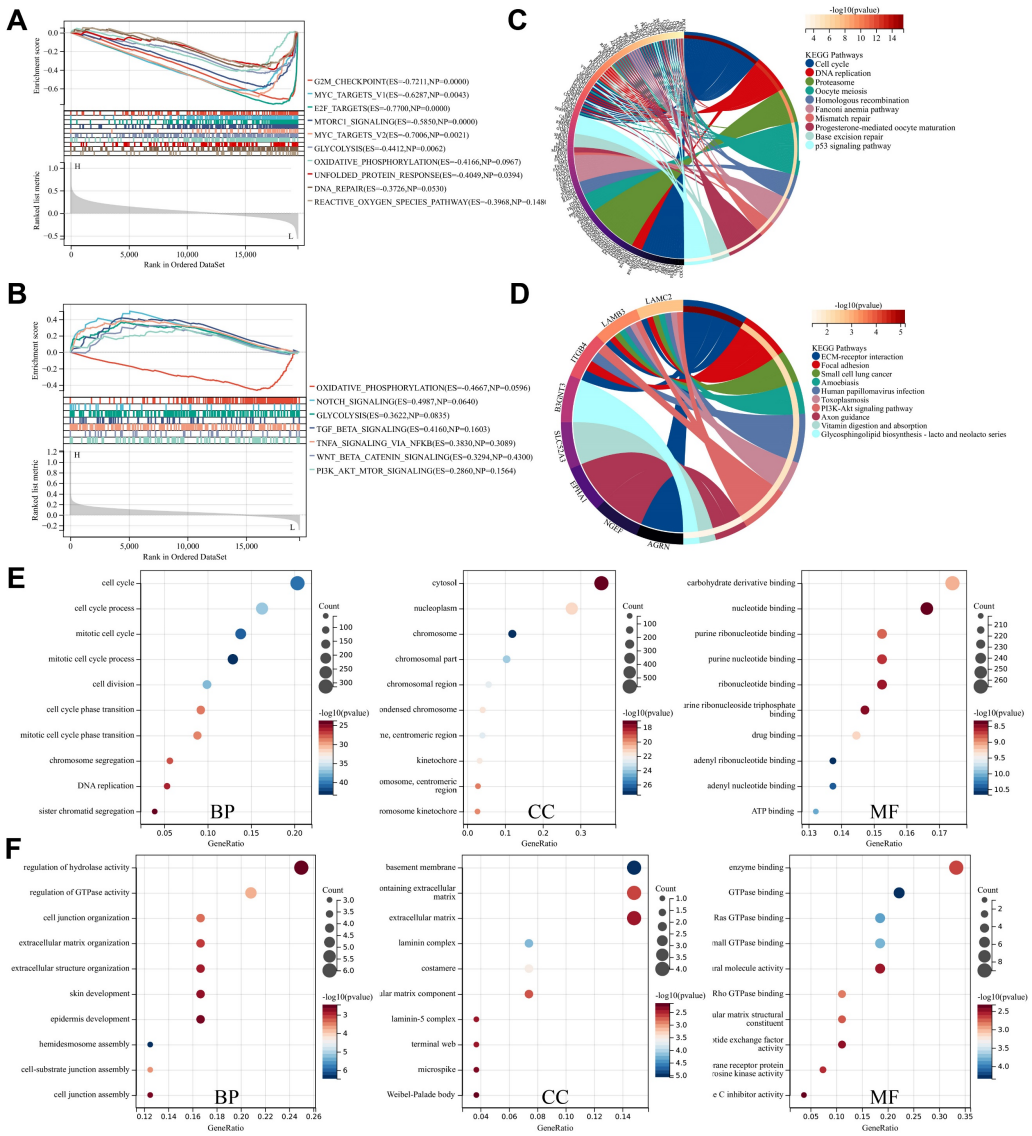
** Enrichment analysis of STARD12 and STARD14 in LUAD**. **(A)** Gene set enrichment analysis (GSEA) for STARD12 and STARD14.** (B)** Enrichment analysis of Kyoto Encyclopedia of Genes and Genomes (KEGG) terms for STARD12 and STARD14 co-expression genes. **(C)** Enrichment analysis of gene ontology (GO) terms for STARD12 and STARD14 co-expression genes.

To deeply explore the roles of STARD12 and STARD14 in LUAD tumorigenesis and progression, we performed GSEA analysis among high and low expression groups of STARD12 and STARD14. The HALLMARK term showed that STARD12 expression was negatively associated with the G2M checkpoint, MYC targets V1 and V2, E2F targets, mTORC1 signaling, glycolysis, oxidative phosphorylation, unfolded protein response process, DNA repair and reactive oxygen species (Figure 6A). Although not statistically different, the analysis showed a trend that STARD14 expression was positively involved in Notch signaling, glycolysis, TGF-BETA signaling, TNFA signaling via NFKB, WNT-BETA-Catenin signaling and PI3K-AKT-MTOR signaling, while negatively associated with oxidative phosphorylation (Figure 6B). Moreover, we adopted the GO term and KEGG pathway investigation of the co-expressed genes of STARD12 as well as STARD14, respectively. The Chordal Graph and bubble chart demonstrated the top 10 messages (Figure 6C-6F). STARD12 co-expressed genes mainly participated in cell cycle and DNA replication, and co-expressed genes of STARD14 were involved in ECM-receptor interaction. The details of the enrichment analysis were summarized in Supplementary Table 4.

### Genetic mutation analysis of STARDs in LUAD

**Figure 7:**
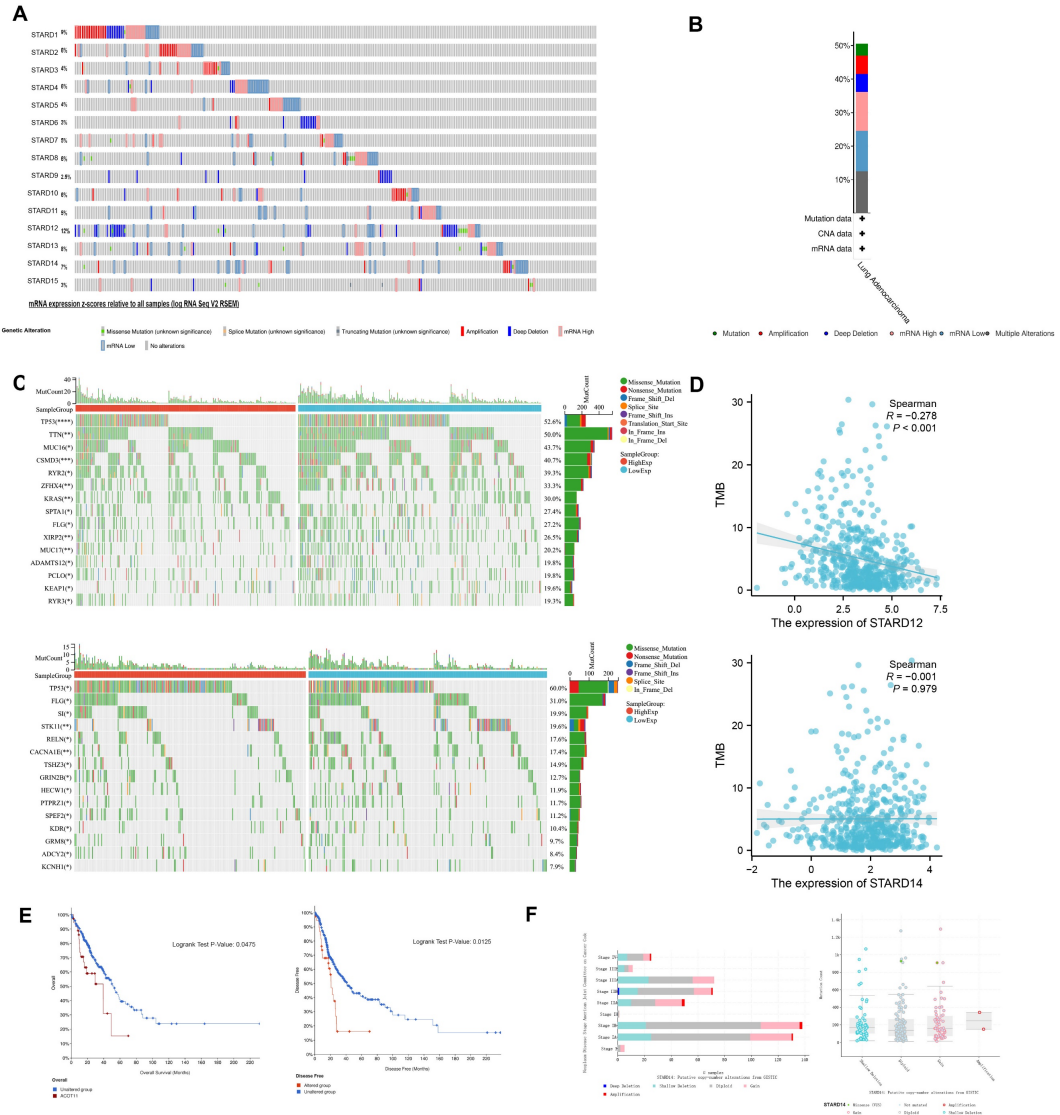
** Genetic mutation analysis of STARDs in LUAD.** (**A**) OncoPrint of STARDs alterations in LUAD cohort. (**B**) Alterations summary in STARDs in LUAD. (**C**) Mutational landscapes of different expression group of STARD12 and STARD14.** (D)** Correlation analysis between TMB and the expression level of STARD12 and STARD14. (**E**) Kaplan-Meier plots comparing OS and disease-free survival (DFS) in LUAD with or without STARD14 alteration. (**F**) STARD14 expression in different CNV groups; Distribution of STARD14 CNV frequency in different stage subgroups.

We conducted an integrated exploration of genomic features of STARDs in LUAD patients via cBioPortal. As presented in Figure 7A-7B, the gene altered in 50.48% of 517 LUAD cases, totally, including amplification, deletion, mRNA high, or mutation. STARD1/12/13/14 were the most frequently altered genes (12%, 9%, 8%, and 7%, respectively). Mutational landscapes found TP53, TTN, MUC16, CSMD3 and RYR2 were the highest frequency mutation genes in the low expression group of STARD12.

In the same time, the high expression group of STARD14 had higher TP53 mutation levels, other significantly different mutated genes included FLG, SI, STK11 and RELN (Figure 7C). As shown in Figure 7D, the correlation analysis between the expression levels of STARD12 and STARD14 and TMB showed a significant negative correlation between the expression levels of STARD12 and TMB. Unfortunately, there was no significant correlation between the expression level of STARD14 and TMB. For clinical attributes, STARD14 alterations were significantly associated with worse OS and DFS (Figure 7E). The detailed distribution and association between the frequency of STARD14 variation and clinical stages in LUAD were presented in Figure 7F, which suggested an early event and high distribution of STARD14 alteration in LUAD.

### Association of STARDs expression with m6A modification in LUAD

**Figure 8:**
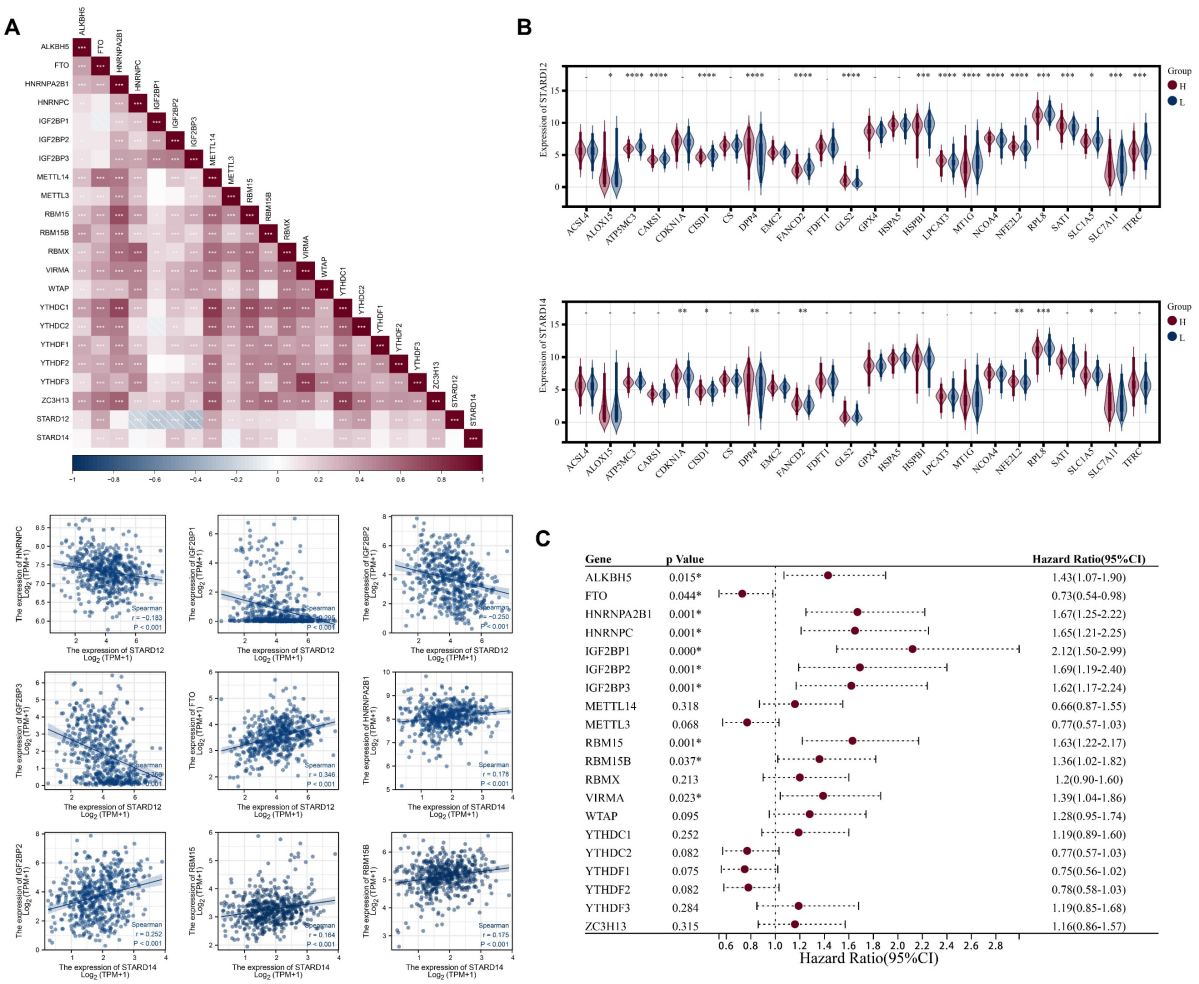
** Correlation analysis of STARD12/14 expression with m6A modification in LUAD**. **(A)** Expression correlation heatmap of STARD12/14 and m6A related genes. Scatter plots showed the detailed association between STARD12/14 and m6A relative genes, including STARD12 with HNRNPC, IGF2BP1, IGF2BP2, IGF2BP3, and FTO, and STARD14 with RBM15 and HNRNPC. **(B)** Violin plots of differential expression of m6A relative genes between high and low STARD12/14 expression groups. **(C)** Forest plot of the prognostic association of m6A related genes with OS. *, *p* < 0.05; **, *p* < 0.01; ***, *p* < 0.001; ****, *p* < 0.0001.

We next explored the association of STARD12/14 expression levels with m6A modification in TCGA-LUAD cohort. The results were shown in Figure 8A-8C. The STARD12 expression was positively associated with ALKBH5, FTO, METTL14, METTL3, RBMX, YTHDC1, YTHDC2, YTHDF2, YTHDF3 and ZC3H13, while negatively associated with HNRNPC, IGF2BP1, IGF2BP2, IGF2BP3 and WTAP (p<0.05). HNRNPC, IGF2BP1, IGF2BP and IGF2BP3 were decreased in high STARD12 expression group, while FTO, METTL14, METTL3, RBM15, YTHDC1, YTHDC2, YTHDF2, YTHDF3 and ZC3H13 were relatively increased (p<0.05). Survival analysis found HNRNPC, IGF2BP1, IGF2BP2, IGF2BP3, YTHDF3, ZC3H13 and ALKBH5 were adverse factors of OS in LUAD patients, and FTO played a protective role. Taken together, HNRNPC, IGF2BP1, IGF2BP2, IGF2BP3 and FTO may be key genes associated with m6A modification, which partially explained the mechanism of its protective role in LUAD. Similarly, we figured out the relation between STARD14 and m6A modification in LUAD. STARD14 expression was positively correlated with most m6A associated genes (p<0.05). FTO, HNRNPA2B1, IGF2BP2, METTL14, RBM15, RBM15B, YTHDC1, YTHDC2, YTHDF2 and ZC3H13 were overexpressed in STARD14 high expression group (p<0.05). Prognostic analysis identified that high expression of HNRNPA2B1, IGF2BP2, RBM15 and RBM15B were associated with worse survival in LUAD. These analyses indicated that m6A modification played an important role in the process of transcriptional expression of STARD14 and was associated with the prognosis of LUAD patients.

### Immune signature of STARDs in LUAD

**Figure 9:**
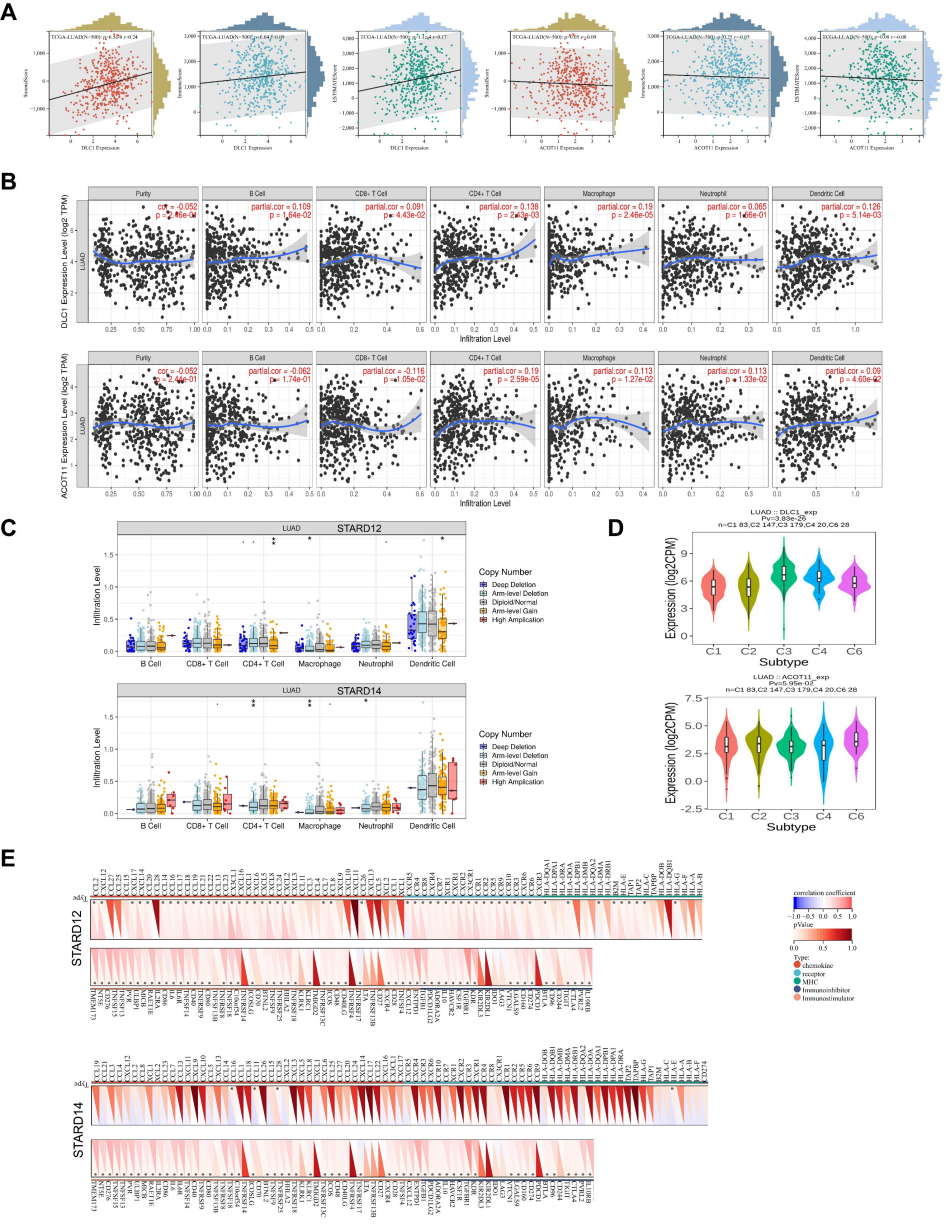
** Immune signature of STARD12/14 in LUAD. (A)** Correlation between STARD12/14 expression and stromal scores, immune scores, as well as ESTIMATE scores. **(B)** Correlation between immune cell infiltration and STARD12/14 expression.** (C)** Correlation between immune cell infiltration and somatic CAN of STARD12/14. (**D**) Correlation between STARD12/14 expression and immune subtypes. (**E**) Correlation between STARD12/14 expression and chemokine, receptor, MHC, immunoinhibitory, immunostimulatory. *p < 0.05, **p < 0.01, ***p < 0.001.

We first calculated the stromal, immune scores and ESTIMATE scores to assess the immune signature of STARD12/14 in LUAD. As shown in Figure 9A, the expression of STARD12 is positively connected with stromal scores, immune scores, as well as ESTIMATE scores in LUAD(p<0.05), while STARD14 level showed negative association with the scores (though p>0.05). Further, we evaluated the correlation between STARD12, STARD14 expression and immune cell infiltration in LUAD patients. The results indicated that the expression level of STARD12 was positively associated with the infiltrating proportion of B cell (r = 0.109, *p* = 1.64E-2), CD8+ T cell (r = 0.091, *p* = 4.42E-2), CD4+ T cell (r = 0.138, *p* = 2.43E-3), macrophage (r = 0.126, *p* = 5.14E-3), while STARD14 expression was negatively related with CD8+ T cell (r =-0.116, *p* = 1.05E-2) (Figure 9B). In addition, STARD12 CNV was significantly correlated with the infiltration proportion of CD4+ T cell, macrophage and DC, and STARD14 CNV also showed correlation with the level of CD4+ T cell and macrophage (Figure 9C). STARD12 expression level was found to play different roles in immunophenotyping C1-C6 in LUAD (Figure 9D). Furthermore, to broaden the understanding of the crosstalk of STARD12 and STARD14 with immune genes, we explored the correlations between STARD12, STARD14 expression level and various immune signatures in LUAD, including immune cell marker genes, chemokine, receptor, MHC, immunoinhibitory, immunostimulatory (Figure 9E and Table 1). In our study, we found that STARD12 expression was significantly associated with the marker genes of tumor infiltrating immune cells (TIICs), including DC, NK cells, Neutrophil cells, Monocyte, B cells, Th2 and Th17 cells. As for the relationship with other immune-related genes, STARD12 expression was positively corelated with chemokines, including CCL2, CXCL12, CXCL14, CXCL17, CCL20, CCL14, CCL16, CCL17, CCL18, CCL19, CCL21, CCL22, CCL13, CCL23, CX3CL1, CXCL16, CXCL1, CXCL6, CXCL5,CXCL8, CCL24, CXCL2, CXCL3, CCL7, CCL8, CCL26, and receptors including CXCR5, CCR4, CCR8, CXCR4, CCR7, XCR1, CXCR1, CXCR2, CX3CR1, CCR1, CCR2, CCR5, CCR9, CCR10, CCR3, CXCR6, CCR6 in LUAD. The expression level of STARD12 also showed positive association with various MHC related genes. In addition, STARD12 was positively associated with immunostimulatory genes, such as T cell costimulatory molecules including ICOS, CD28, CD80 and CD86. Contrast to STARD12, STARD14 was involved in the suppression of anti-tumor immune. STARD14 expression was negatively associated with the marker genes of B cells, CD8 + T cells, while positively related with Treg cells and M2 Macrophage. Majority of chemokine, receptor, MHC were negatively associated with STARD14 expression. Both STARD12 and STARD14 exhibited positive relevance to star immune checkpoints, such as PDCD1, PDCD1LG2, LAG3, and CTLA4.

### Association of STARDs expression with ferroptosis in LUAD

**Figure 10:**
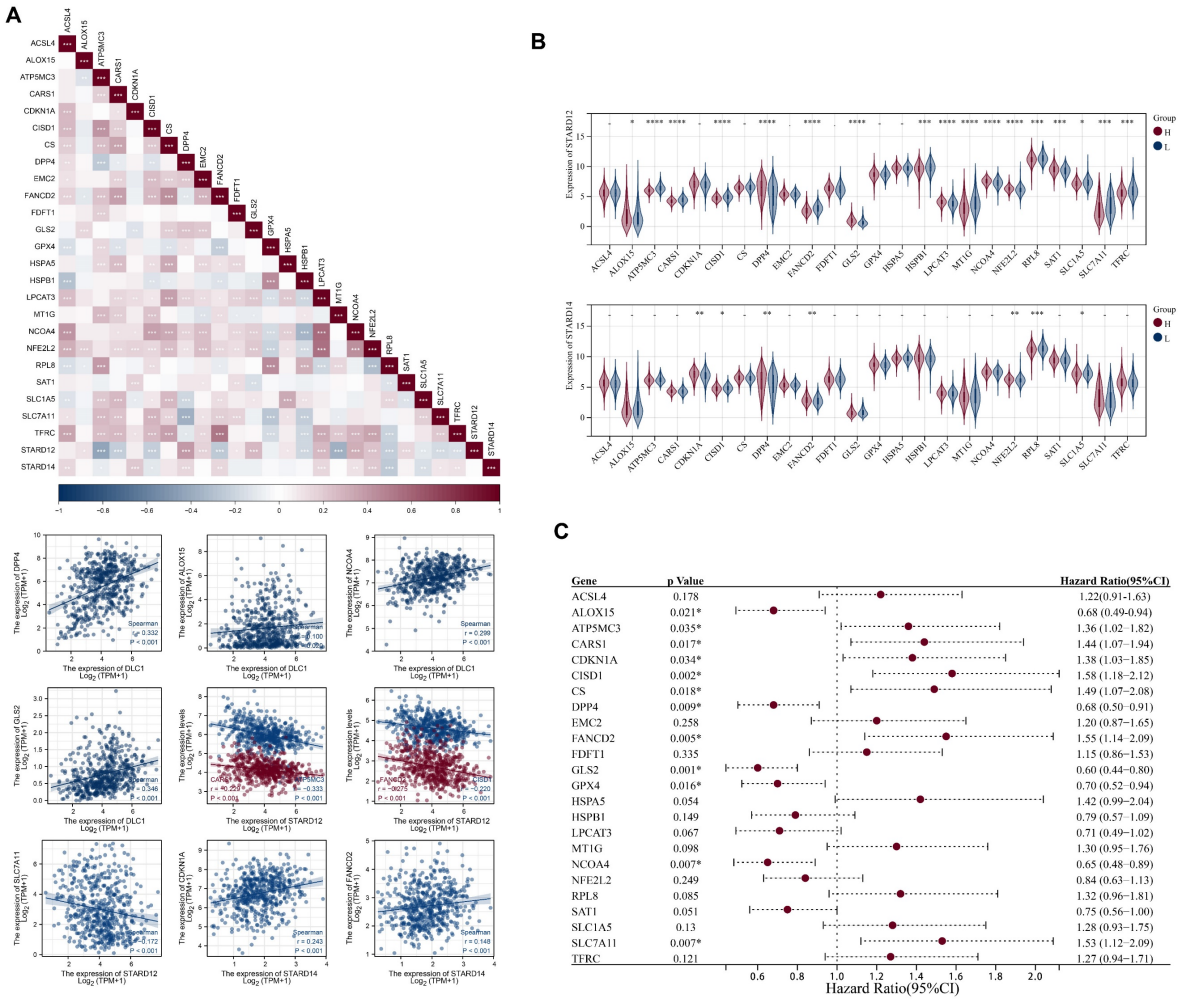
** Correlation analysis of STARD12/14 expression with ferroptosis in LUAD. (A)** Expression correlation heatmap of STARD12/14 and ferroptosis related genes. Scatter plots showed the detailed association between STARD12/14 and ferroptosis relative genes, including STARD12 with ALOX15, DPP4, GLS2, NCOA4, ATP5MC3, CARS1, CISD1, FANCD2 and SLC7A11, and STARD14 with CDKN1A and FANCD2. **(B)** Violin plots of differential expression of ferroptosis relative genes between high and low STARD12/14 expression groups. **(C)** Forest plot of the prognostic association of m6A related genes with OS. *, *p* < 0.05; **, *p* < 0.01; ***, *p* < 0.001; ****, *p* < 0.0001.

Ferroptosis is closely associated with lipid metabolism, especially fatty acid metabolism. To explore the association of STARD12/14 with ferroptosis, we selected 25 ferroptosis related genes based on the literature and performed correlation, differential expression and prognosis analysis in TCGA-LUAD cohort (Figure 10A-10C). STARD12 was significantly positively correlated with ALOX15, DPP4, EMC2, FDFT1, GLS2, LPCAT3, NCOA4, NFE2L2 and SAT1, but negatively correlated with ATP5MC3, CARS1, CISD1, FANCD2, HSPB1, MT1G, RPL8, SLC1A5, SLC7A11 and TFRC (p<0.05). The expression difference analysis was generally consistent with the correlation analysis (p<0.05). Further survival analysis found that patients with ALOX15, DPP4, GLS2 and NCOA4 high expression had a longer OS, while patients with ATP5MC3, CARS1, CISD1, FANCD2 and SLC7A11 high expression had a worse survival. Taken together, we found that STARD12 may be closely associated with iron death by regulating the expression of the above nine genes and ultimately affect the prognosis of LUAD patients. STARD14 was significantly positively correlated with ACSL4, CDKN1A, CS, DPP4, FANCD2, LPCAT3, NFE2L2 and TFRC, and negatively correlated with CISD1, RPL8 and SLC1A5 (p<0.05). CDKN1A, DPP4, FANCD2 and NFE2L2 was significantly over expressed in the STARD14 high expression group, whereas CISD1, RPL8 and SLC1A5 was under expressed (p<0.05). Finally combined with survival analysis identified CDKN1A and FANCD2 as the key genes of STARD14 playing a cancer promoting role in LUAD.

### Quantitative real-time PCR of STARDs in LUAD

**Figure 11:**
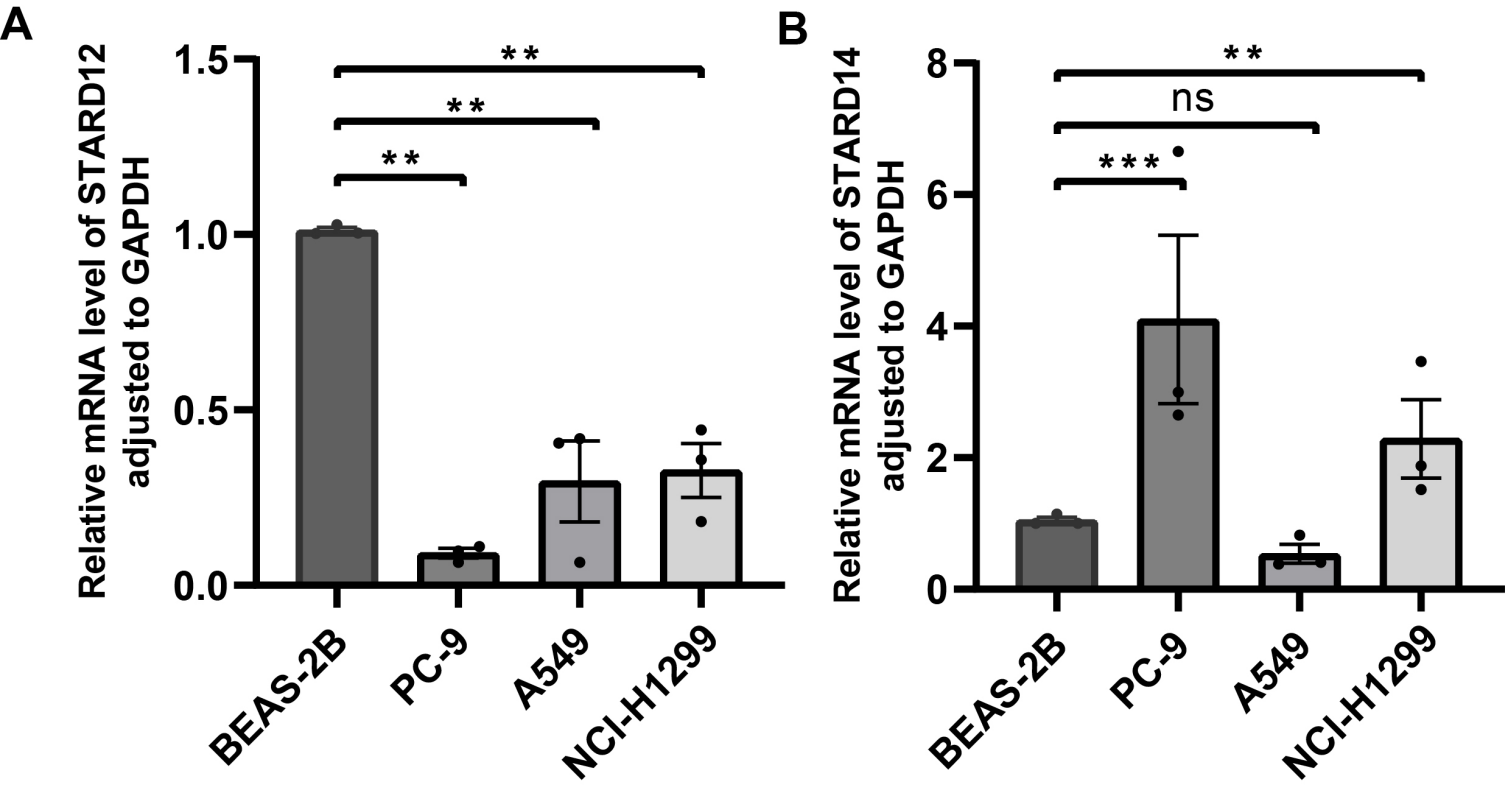
** Validation of the STARD12/14 expression in LUAD via RT-qPCR. (A)** Differential expression of STARD12 in LUAD cell lines and human lung epithelial cell line.** (B)** Differential expression of STARD14 in LUAD cell lines and human lung epithelial cell line. *p < 0.05, **p < 0.01, ***p < 0.001.

**Figure 12:**
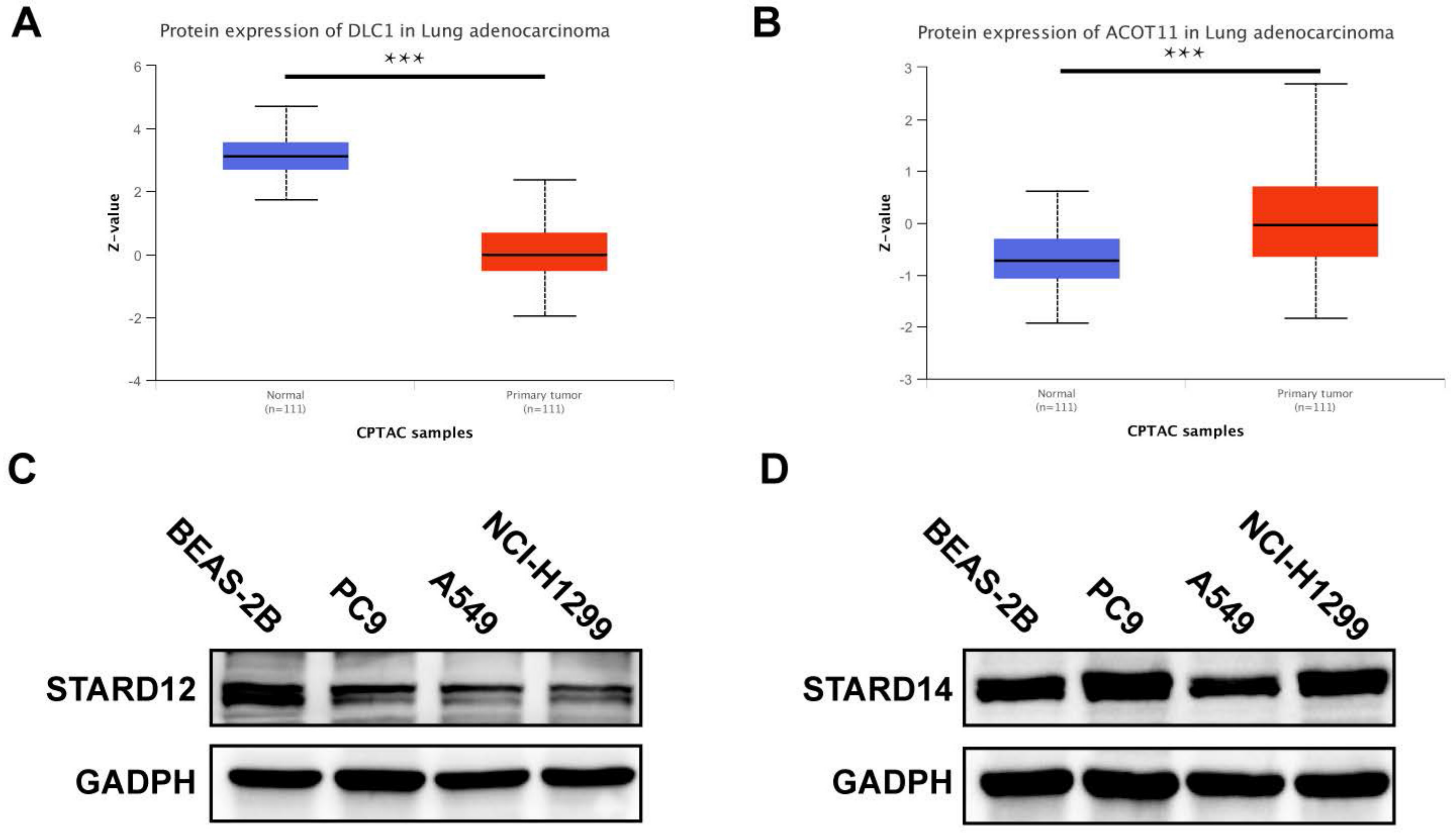
** Protein expression level of STARD12/14 expression in LUAD via CPTAC and western blot.** (A) STARD12 protein level in LUAD tissues compared with the normal. (B) STARD14 protein level in LUAD tissues compared with the normal. (C) Differential expression level of STARD12 in LUAD cell lines and human lung epithelial cell line. (D) Differential expression level of STARD14 in LUAD cell lines and human lung epithelial cell line. *p < 0.05, **p < 0.01, ***p < 0.001.

To validate the STARD12 and STARD14 expression in LUAD, we examined the mRNA levels of STARD12 and STARD14 in cell lines (BEAS-2B, PC-9, A549 and NCI-H1299). RT-qPCR data indicated that STARD12 mRNA expression was significantly reduced in PC-9, A549 and NCI-H1299 cell lines, compared with normal human lung epithelial cell line BEAS-2B (Figure 11A). As shown in Figure 12B, STARD14 was found to overexpress in PC-9 and NCI-H1299, while without statistical difference in A549 cell line.

### Protein expression level of STARDs in LUAD

We further used CPTAC database and Western blot analysis to detect the expression level of STARD12 and STARD14 in LUAD. As shown in Figure 12A-B, the protein levels of STARD12 were lower in LUAD compared to normal tissues; and STARD14 is significantly overexpressed in LUAD tissue. Western blot results showed that compared with human normal lung epithelial cell line BEAS-2B, STARD12 was significantly lower expressed in PC9, A549 and NCI-H1299 cell lines (Figure 12C); And STARD14 is significantly overexpressed in LUAD cell lines, including PC9 and NCI-H1299, but decreased in A549 (Figure 12D).

## Discussion

Based on metabolic changes, a new concept of "metabolic reprogramming" has been proposed in the oncology field (20). In addition to regulating nutrients uptake, it is considered to account for cancer-causing mutations and metabolization-driven gene regulation and interact with the tumor microenvironment (TME) 20. LTPs are implicated in cell transformation and malignant phenotype 21. The STARDs we discussed are important members of LTPs. Although previous studies have confirmed STARDs could play roles in tumor proliferation and metastasis 6, the potential functions of STARDs in LUAD is not clarified yet. Thus, our research decided to make a comprehensive analysis in terms of prognostic value and immune signature of STARDs in patients with LUAD.

We examined the transcriptional expression of STARDs in LUAD and normal samples using Oncomine and UALCAN databases, and revealed differential expression in majority of STARDs. STARD5/10/14 were overexpressed and STARD4/7/8/11/12/13 were down-regulated in LUAD patients compared with the normal. Then we analyzed the relationship between STARDs and clinicopathological parameters in LUAD, and found that the mRNA expressions of STARD5/12/14 were positively or negatively correlated with cancer stage and lymph node stage of LUAD patients. Combined the further survival analysis, we suggested STARD12 and STARD14 as the most potential biomarker of STARDs worthy of further exploration and validation in LUAD.

STARD12, also known as Deleted in Liver Cancer-1(DLC-1) protein, was considered a tumor suppressor in liver cancer, breast cancer, gastric cancer, and renal cell carcinoma 22. STARD12 could suppress tumor cell invasion by RhoGAP-dependent and independent mechanisms. Evidence suggested that the initiation domain and SAM domain-binding peptides, including TNS3 or PTEN C2, were also involved in the inhibitive activities 23, 24. Besides, studies revealed STARD12 could regulate the immunomodulation of hMSCs via interaction with Notch1 25 and degrade angiogenesis by reducing VEGF in epithelial cells 26. Recently, its potential function in lung cancer has been explored initially. Zhang et al. 27 suggested that STARD12 could exert anti-metastasis effect on NSCLCs by regulating the TGF-β1-induced CD105 of STARD12-RhoA-Rock1 pathway. Besides, they determined the effect was implemented by inhibiting SMAD3 linker region phosphorylation as well as the protein nuclear translocation. In our study, relatively lower expression of STARD12 was observed in LUAD patients, which was consistent with the complementary results of immunohistochemical staining at the protein level Li et al. 28. Moreover, STARD12 low expression was significantly associated with shorter OS of LUAD patients, suggesting that STARD12 may be a prognostic biomarker for LUAD.

STARD14, also known as Acyl-CoA Thioesterase 11 (ACOT11), is a member of the type II Acyl-CoA thioesterase family in addition to belonging to thioesterase group of STARD family 6. STARD14 is active in catalyzing the hydrolysis of activated fatty acids. Specifically, STARD14 can convert fatty acyl COAs into the non-esterified fatty acids, which could participate in the regulation of physiology by maintaining the intracellular levels of fatty acyl CoAs, FFAs and CoASH 29 30. In addition, the STARD domain of STARD14 may interact with the hotdog domain to mediate intracellular functions, including lipid trafficking, lipid metabolism, and transmission of cellular signals 5. STARD14 was revealed to harbor heterogeneous roles in different tumors: on the one hand, STARD14 is downregulated in clear cell renal cell carcinoma and colon cancer and considered as a protective biomarker for tumorigenesis 31 32; on the other hand, STARD14 expression is higher in NSCLC and associated with poor prognosis in LUSC patients 33. Liang et al. 34 found that STARD14 can promote cell proliferation, migration, invasion and epithelial mesenchymal transition in lung adenocarcinoma, and can also inhibit cell apoptosis and cell cycle arrest. Molecular mechanism study found that STARD14 may promote tumor progression via directly act on CSE1L, and associated with several multiple tumor-related signaling, including Wnt/ β - catenin signaling, PI3K / Akt signaling, Cdc42 signaling, and SAPK / JNK signaling 34. In our study, higher mRNA expression of STARD14 was identified in LUAD paralleled with the non-tumor samples. Survival analysis results found STARD14 level was remarkably related with shorter OS of LUAD patients.

Functional enrichment analysis identified that, in addition to exhibiting important roles in lipid metabolism, STARDs were found to regulate fibroblast migration and cytoskeletal tissues, including focal adhesion, contractile actin filament bundles, stress fibers, endosome membrane and other cellular components, which allowed STARDs to obtain the ability to assist tumor-infiltrating cells to migrate 35, 36. Besides, STARDs were also closely involved in TGF- β receptor signaling pathway. TGF-β pathway could induce cell cycle arrest with apoptosis and EMT. In addition, it enabled to induce an immunosuppressive TME, such as promoting Treg subset differentiation, and inhibiting the antigen-presenting function of dendritic cells, thereby leading to immune escape of tumor cells 37-39. Given all of it, our results suggested that STARDs could contribute to tumorigenesis and tumor immune microenvironment probably by the TGF- β pathway. GSEA analysis identified that STARD12 was negatively associated with cell cycle, especially via regulating the G2M checkpoint, MYC targets V1 and V2, E2F targets, DNA repair and mTORC1 signaling. In addition, glycolysis and oxidative phosphorylation activity was downregulated in STARD12 low-expression group. Functional enrichment analysis found that STARD14 co-expressed genes were mainly involved in ECM receptor interaction. GSEA analysis found positive correlation between STARD14 expression and Notch signaling, glycolysis, TGF- β signaling, TNF-α via NFkB signaling, Wnt/β- catenin signaling and PI3K-Akt-mTOR signaling pathways, while negative correlation with oxidative phosphorylation activity.

Cancer cells have the ability of metabolic reprogramming to continuously proliferate in response to the energy and substrate needs 3 4. Interactions between metabolic pathways and signaling pathways lead to metabolic reprogramming. In addition, intrinsic (genomic alterations, e.g., copy number abnormalities, somatic mutations, etc.) and extrinsic factors (nutrient intake, medicine applications, and tumor microenvironment) also contribute to the cancer metabolic reprogram 4. Enrichment analysis identified that both STARD12 and STARD14 are involved in regulating metabolic pathways including lipid metabolism, glycolysis, oxidative phosphorylation, and closely related to cancer-related signaling pathways. Next, we further explored the epigenetic features of STARD12/14 in LUAD. Genetic analysis results revealed STARD12/14 as the most frequently altered genes in STARDs (9% and 7%, respectively) and STARD14 alterations were associated with worse OS and DFS in LUAD patients. Moreover, significant relevance between STARD12/14 expression level, CNAs, and immune infiltration. Mutational landscapes found that higher TP53 mutation existed top in both low-STARD12 and high-STARD14 group. Wild type TP53 can maintain metabolic homeostasis by regulating the expression of metabolism related genes or the activity of enzymes, such as inhibiting glycolysis, enhancing mitochondrial oxidative phosphorylation, inhibiting fatty acid synthesis, while TP53 mutations are involved in the metabolic reprogramming to promote tumor development 40. In addition, TP53 mutations were associated with immunoregulatory effects. Wild type TP53 could induce CCL2 production and mediates the activation and recruitment of natural killer (NK) cells to the TME 41, while TP53 mutations were correlated with the immunosuppressive M2 phenotype of tumor associated macrophages (TAMs) 42. TMB can indirectly reflect the ability and degree of tumors to produce new antigens, and predict the efficacy of immunotherapy for various tumors 43. The correlation analysis results found a significant negative association between STARD12 expression and TMB level. Patients with high expression of STARD12 may have better reactivity to immune checkpoint inhibitors, and the predictive value of STARD12 can be further explored in the immunotherapy cohort in the future. m6A modification is the most prevalent RNA methylation modification, which is closely related to carcinogenesis and metastasis, lipid metabolism, and DNA damage repair 44. Combined with expression and prognosis analysis, HNRNPC, IGF2BP1, IGF2BP2, IGF2BP3, and FTO may be key genes associated with m6A modification, which may define the protective ability of STARD12 in LUAD. We also found the dependence of STARD14 on RNA methylation levels in LUAD, including the correlation with HNRNPA2B1, IGF2BP2, RBM15 and RBM15, which could participate in the tumor progression of LUAD. Li et al. 45 identified RBM15 and HNRNPC as oncogenes in LUAD, and constructed a prognostic risk score model based on KIAA1429, RBM15 and HNRNPC for LUAD patients. Genes encoding the IGF2BP family, including IGF2BP 1, IGF2BP2 and IGF2BP3, were widely overexpressed and associated with poor prognosis in a variety of human malignancies as RNA stabilizers 46. For example, Jia et al. 47 found high transcription level of IGF2BP2 and the association with poor prognosis in LUAD. Further, they suggested that IGF2BP2 could exert its oncogenic effect through the competitive action of WT1-AS with miR-200a based on a c-Myc-associated ceRNA network analysis. IGF2BP2 also contributed to tumorigenesis by regulating cancer metabolism; IGF2/PI3K/AKT pathway clearly played important signal in the proliferation, invasion, and metastasis of LUAD 48. IGF2BPs exerted radio resistance in LUAD patients via upregulating VANGL1, while ubiquitination of IGF2BPs can inhibit tumor growth 49, 50. We suggested that the protective or promoting roles in LUAD patients played by STARD12 and STARD14 may be associated with epigenetic regulation.

TME refers to the surrounding microenvironment in which tumor cells exist. Immune cells and stromal cells are the two major types of non-tumor components, which play critical roles in regulating malignant progression and modulating responses to therapies 51. Metabolic reprogramming of tumor cells not only plays a key role in maintaining tumorigenesis but can also affect other cell types in the tumor microenvironment, especially immune cells 52. STARDs are involved in tumor metabolic reprogramming, the correlation between STARDs and TME acquires further exploration. In our study, STARD12 high-group had higher stromal scores, immune scores and ESTIMATE scores in LUAD, while STARD14 showed relatively low scores. We found the expression of STARD12 was positively correlated with the abundance of infiltrating immune cells, including B cells, and CD4+ T cells, CD8+T cells, macrophages, and dendritic cells, which enhanced the anti-tumor immune response. STARD12 was positively associated with an overwhelming majority of chemokine, receptor and MHC related genes. These genes are involved in the upregulation of antigen presentation, processing and the recruitment of TIICs such as antigen-presenting cells, CD4+ T cells, CD8+ T cells and TH17 cells. The expression of STARD14 was bound with lower infiltration of CD8+T cells as well as marker genes expression, which indicated inhibitory of anti-tumor immunity. In addition, the STARD14 expression was positively association with the marker genes of Treg cells and M2 Macrophage. Most chemokine, receptor and MHC related genes were negatively correlated with the expression of STARD14, but CCL28 was positively associated with the STARD14 expression level. The hypoxic environment of tumors could induce CCL28 expression, which promoted the infiltration of Treg cells into tumor 53. Tregs can suppress costimulatory signal CD80/CD86 expression via CTLA-4, as well as secrete the immunosuppressive cytokine TGF-β and IL-10 to inhibit T cell activation and response, thus mediating tumor immune escape 54, 55. TAMs are major components of immune cells in the TME of solid tumors, and the M2 phenotype contributes to tumorigenesis and promotes malignant progression, including angiogenesis and metastasis 56. Currently, monoclonal antibodies for strengthening T-cell immunity have been a dramatic shift in anti-tumor treatment 57. Specific antibodies targeting PD-1 and PD-L1 have been the first-line treatment strategy for patients with metastatic NSCLC 58, which had greatly improved the long-term survival of LUAD patients. However, investigators found there were still a subset of patients who responded poorly to immunotherapy. These patients usually presented immunologically with poor T-cell infiltration 59. The results suggested that highly expressed STARD14 tended to lack the expression of chemokines, T cell infiltrations, and related markers in LUAD. Overcoming the non-T cell inflammatory tumor microenvironment could theoretically improve the efficacy of cancer immunotherapy 60. STRAD14 were found to possess remarkable associations with immune checkpoints, including CTLA4 as well as PD-L2, which would be a target for boosting immunotherapy effects in LUAD. Our study provided detailed immune information of STARD12 and STARD14 in LUAD cancers, which would be a target for boosting immunotherapy effects in LUAD. Further studies were needed to elucidate whether STARD12 and STARD14 could act as key players in mediating immune therapy.

Ferroptosis refers to an iron dependent oxidative form of regulated cell death and plays dual roles in tumorigenesis by modulating tumor microenvironment and tumor immunity 61. The expression pattern, prognostic value and mechanistic analysis of ferroptosis related genes have been initially explored in LUAD 62-64. In our study, iron death was more closely linked to the protective role of STARD12, which may participate in the regulation of nine ferroptosis associated genes including ALOX15, DPP4, GLS2, NCOA4, ATP5MC3, CARS1, CISD1, FANCD2 and SLC7A1 to suppress tumor progression of LUAD. Similarly, our integrated analysis identified CDKN1A and FANCD2 as the key genes of STARD14 playing a cancer promoting role in LUAD. We suggested that STARD14 may achieve the regulation of ferroptosis in LUAD by promoting the expression of CDKN1A and FANCD2, ultimately contributing to tumor progression.

There were some limitations in our study. Firstly, our work was a bioinformatics analysis and the results were mainly retrieved from online databases. Secondly, there was a lack of more mechanism exploration in vitro and in vivo, so further experimental evidences were required to prove our conclusions in our work.

## Conclusion

In conclusion, this study provided multilevel evidences for potential therapeutic and prognostic roles of STARDs in LUAD. STARD12 was served as a protective gene in LUAD, while STARD14 was an oncogene, which were associated with epigenetic regulation, immune infiltration as well as ferroptosis.

## Supplementary Material

Supplementary Table 1 Primer sequences of STARD12/14 and GAPDH; Supplementary Table 2 The significant differences of transcriptional expression of STARDs between diverse types of LUAD and normal samples (Oncomine); Supplementary Table 3 Univariate and multivariate Cox analysis of STARD12 and STARD14 in LUAD patients; Supplementary Table 4 Enrichment analysis results of STARDs in LUAD; Supplementary Figure 1 Pathological histological subgroup expression analysis of STARDs in LUAD; Supplementary Figure 2 Survival analysis on DFS of STARDs in LUAD; Supplementary Figure 3 Survival analysis on PFS of STARDs in LUAD; Supplementary Figure 4 Survival analysis on DSS of STARDs in LUAD.

## Figures and Tables

**Table 1:** Correlation analysis between STARD12/14 and immune cell marker genes in TIMER

Description	Gene markers	STARD12				STARD14			
		None		Purity		None		Purity	
		rho	*p*	rho	*p*	rho	*p*	rho	*p*
B cell	CD19	0.034	4.43E-01	0.012	7.86E-01	-0.063	1.51E-01	-0.114	1.14E-02*
	MS4A1	0.156	3.81E-04*	0.145	1.20E-03*	-0.051	2.48E-01	-0.094	3.73E-02*
	CD79A	0.029	5.18E-01	0.012	7.98E-01	-0.082	6.21E-02	-0.126	5.10E-03*
CD8 + T Cell	CD8A	-0.059	1.84E-01	-0.088	5.12E-02	-0.076	8.63E-02	-0.113	1.24E-02*
	CD8B	-0.121	5.84E-03*	-0.147	1.09E-03*	-0.066	1.37E-01	-0.093	3.88E-02*
	IL2RA	-0.010	8.27E-01	-0.040	3.76E-01	0.001	9.83E-01	-0.013	7.71E-01
Tfh	CXCR3	-0.016	7.10E-01	-0.054	2.28E-01	0.132	2.63E-03*	0.114	1.17E-02*
	CXCR5	0.116	8.51E-03*	0.103	2.26E-02*	0.055	2.10E-01	0.028	5.32E-01
	ICOS	0.077	8.20E-02	0.045	3.19E-01	0.021	6.28E-01	-0.016	7.26E-01
Th1	IL12RB1	0.026	5.60E-01	-0.004	9.26E-01	0.061	1.65E-01	0.046	3.09E-01
	CCR1	0.118	7.46E-03*	0.093	3.95E-02*	0.023	6.09E-01	0.013	7.76E-01
	CCR5	0.097	2.79E-02*	0.070	1.18E-01	0.037	4.04E-01	0.014	7.52E-01
Th2	CCR4	0.313	3.54E-13*	0.305	4.29E-12*	0.036	4.12E-01	0.009	8.44E-01
	CCR8	0.131	2.98E-03*	0.111	1.37E-02*	0.060	1.72E-01	0.045	3.20E-01
	HAVCR1	0.051	2.49E-01	0.044	3.27E-01	0.134	2.40E-03*	0.141	1.69E-03*
Th17	IL21R	0.014	7.45E-01	-0.030	5.09E-01	0.104	1.79E-02*	0.087	5.42E-02
	IL23R	0.115	8.84E-03*	0.111	1.38E-02*	0.049	2.70E-01	0.029	5.25E-01
	CCR6	0.361	2.95E-17*	0.360	1.43E-16*	0.086	5.14E-02	0.066	1.43E-01
Treg	FOXP3	0.023	6.07E-01	-0.012	7.91E-01	0.131	3.00E-03*	0.112	1.27E-02*
	NT5E	0.027	5.45E-01	0.016	7.20E-01	0.121	5.93E-03*	0.102	2.38E-02*
	IL7R	0.292	1.36E-11*	0.289	5.76E-11*	-0.005	9.15E-01	-0.035	4.39E-01
M1 Macrophage	NOS2	0.212	1.25E-06*	0.210	2.58E-06*	0.157	3.48E-04*	0.147	1.05E-03*
	IRF5	0.002	9.66E-01	-0.015	7.47E-01	0.306	1.21E-12*	0.301	8.14E-12*
	PTGS2	0.109	1.34E-02*	0.113	1.24E-02*	-0.005	9.10E-01	-0.021	6.39E-01
M2 Macrophage	CD163	0.158	3.11E-04*	0.148	9.84E-04*	0.042	3.41E-01	0.040	3.71E-01
	MRC1	0.360	3.10E-17*	0.352	7.84E-16*	0.075	8.82E-02	0.076	9.09E-02
	CD209	0.157	3.58E-04*	0.151	7.93E-04*	0.032	4.71E-01	0.021	6.47E-01
TAM	CCL2	0.093	3.51E-02*	0.066	1.41E-01	0.045	3.09E-01	0.020	6.57E-01
	CD86	0.099	2.41E-02*	0.078	8.27E-02	0.002	9.71E-01	-0.014	7.58E-01
	CD68	0.062	1.59E-01	0.047	2.99E-01	0.140	1.40E-03*	0.144	1.31E-03*
Monocyte	CD14	-0.022	6.23E-01	-0.045	3.14E-01	0.059	1.81E-01	0.057	2.03E-01
	CD33	0.179	4.52E-05*	0.158	4.31E-04*	0.049	2.71E-01	0.046	3.08E-01
	ITGAX	0.144	1.02E-03*	0.137	2.23E-03*	0.149	6.69E-04*	0.148	9.64E-04*
Natural killer cell	B3GAT1	0.235	6.54E-08*	0.225	4.57E-07*	0.046	3.01E-01	0.036	4.21E-01
	KIR3DL1	0.016	7.20E-01	0.002	9.71E-01	-0.023	6.00E-01	-0.035	4.35E-01
	CD7	-0.176	5.83E-05*	-0.207	3.51E-06	0.075	8.77E-02	0.060	1.84E-01
Neutrophil	FCGR3A	0.007	8.79E-01	-0.015	7.41E-01	0.049	2.63E-01	0.040	3.78E-01
	CD55	0.439	1.10E-25*	0.447	1.22E-25*	0.022	6.19E-01	0.010	8.30E-01
	ITGAM	0.178	4.76E-05*	0.163	2.85E-04*	0.191	1.23E-05*	0.192	1.78E-05*
Dendritic cell	CD1C	0.419	2.60E-23*	0.406	6.13E-21*	0.049	2.65E-01	0.039	3.91E-01
	THBD	0.391	2.81E-20*	0.385	7.70E-19*	0.056	2.02E-01	0.059	1.89E-01
	NRP1	0.332	1.09E-14*	0.322	2.42E-13*	0.042	3.41E-01	0.035	4.38E-01

Adopted purity adjustment, *p<0.05

## Abbreviations

ACOT11: Acyl-CoA Thioesterase 11
CNA: Copy number alteration
CTLA4: Cytotoxic T-Lymphocyte-Associated Antigen 4
DLC-1: Deleted in Liver Cancer-1
GAPDH: glyceraldehyde 3-phosphate dehydrogenase
GSEA: Gene Set Enrichment Analysis
GTT1: Glutathione S-transferase 1
KEGG: Kyoto Encyclopedia of Genes and Genomes
LAG3: Lymphocyte activation gene 3 protein
LTPs: Lipid-transfer proteins
LUAD: lung adenocarcinoma
LUSC: lung squamous cell carcinoma
NSCLC: non-small cell lung cancer
PD-1: programmed cell death receptor1
PD-L1: programmed cell death1 ligand1
PD-L2: Programmed cell death 1 ligand 2
RT-qPCR: Reverse transcription-quantitative polymerase chain reaction
STARDs: START domain-containing proteins
TCGA: The Cancer Genome Atlas
TGF-β: transforming growth factor beta
TME: tumor microenvironment
