# Supplementary figures and images for: STARD12/14 are diagnostic and prognostic biomarkers of lung adenocarcinoma associated with epigenetic regulation, immune infiltration and ferroptosis
Source: Int J Med Sci. 2023 Sep 11;20(11):1427–47. doi: 10.7150/ijms.84566 (PMC10542189; doi:10.7150/ijms.84566)

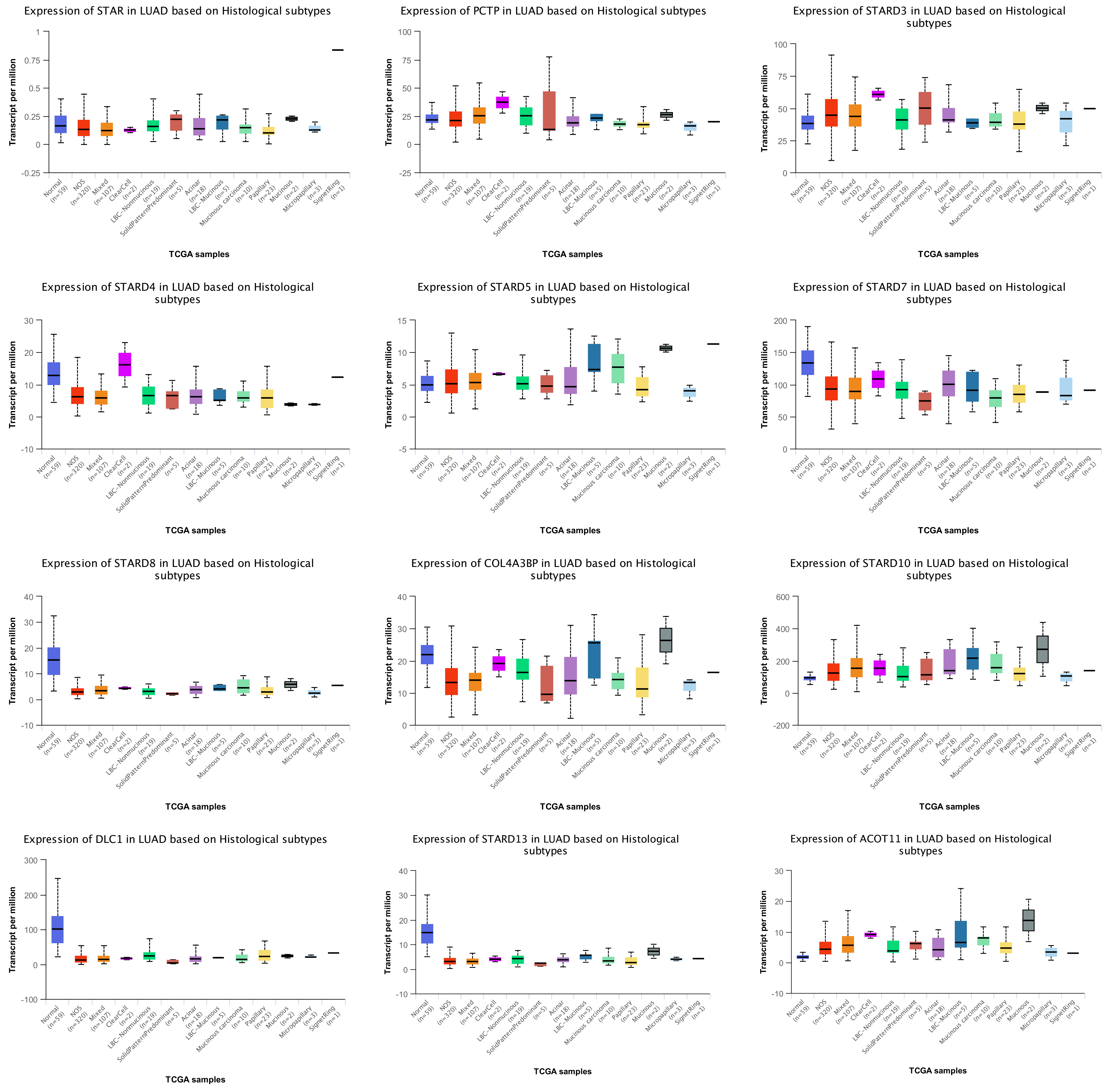

Supplement: Supplementary file 1 — Supplementary Table 1 Primer sequences of STARD12/14 and GAPDH; Supplementary Table 2 The significant differences of transcriptional expression of STARDs between diverse types of LUAD and normal samples (Oncomine); Supplementary Table 3 Univariate and multivariate Cox analysis of STARD12 and STARD14 in LUAD patients; Supplementary Table 4 Enrichment analysis results of STARDs in LUAD; Supplementary Figure 1 Pathological histological subgroup expression analysis of STARDs in LUAD; Supplementary Figure 2 Survival analysis on DFS of STARDs in LUAD; Supplementary Figure 3 Survival analysis on PFS of STARDs in LUAD; Supplementary Figure 4 Survival analysis on DSS of STARDs in LUAD. [file ijmsv20p1427s1.zip › Supplementary materials/Supplementary Figure 1.jpg]

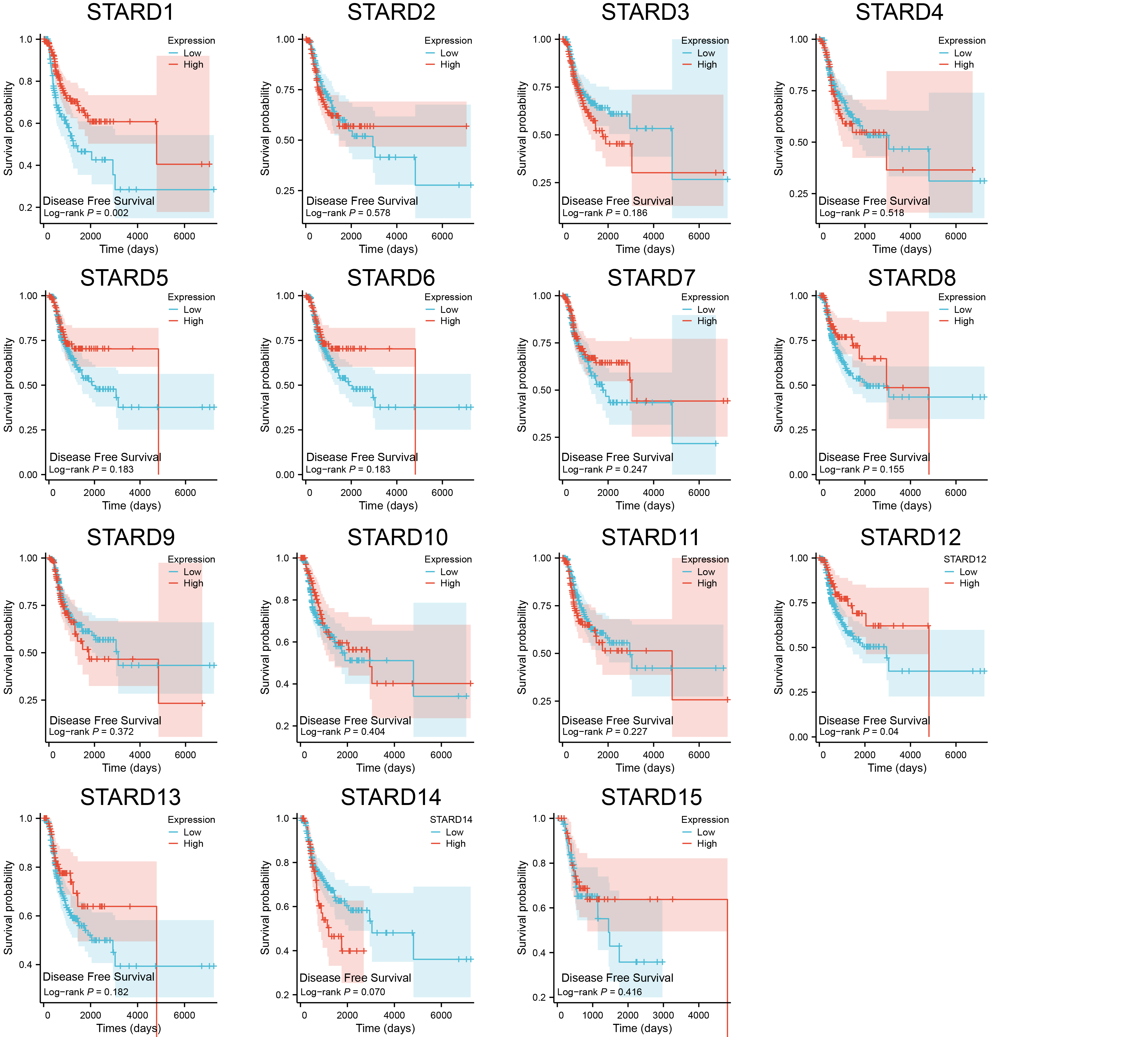

Supplement: Supplementary file 1 — Supplementary Table 1 Primer sequences of STARD12/14 and GAPDH; Supplementary Table 2 The significant differences of transcriptional expression of STARDs between diverse types of LUAD and normal samples (Oncomine); Supplementary Table 3 Univariate and multivariate Cox analysis of STARD12 and STARD14 in LUAD patients; Supplementary Table 4 Enrichment analysis results of STARDs in LUAD; Supplementary Figure 1 Pathological histological subgroup expression analysis of STARDs in LUAD; Supplementary Figure 2 Survival analysis on DFS of STARDs in LUAD; Supplementary Figure 3 Survival analysis on PFS of STARDs in LUAD; Supplementary Figure 4 Survival analysis on DSS of STARDs in LUAD. [file ijmsv20p1427s1.zip › Supplementary materials/Supplementary Figure 2.jpg]

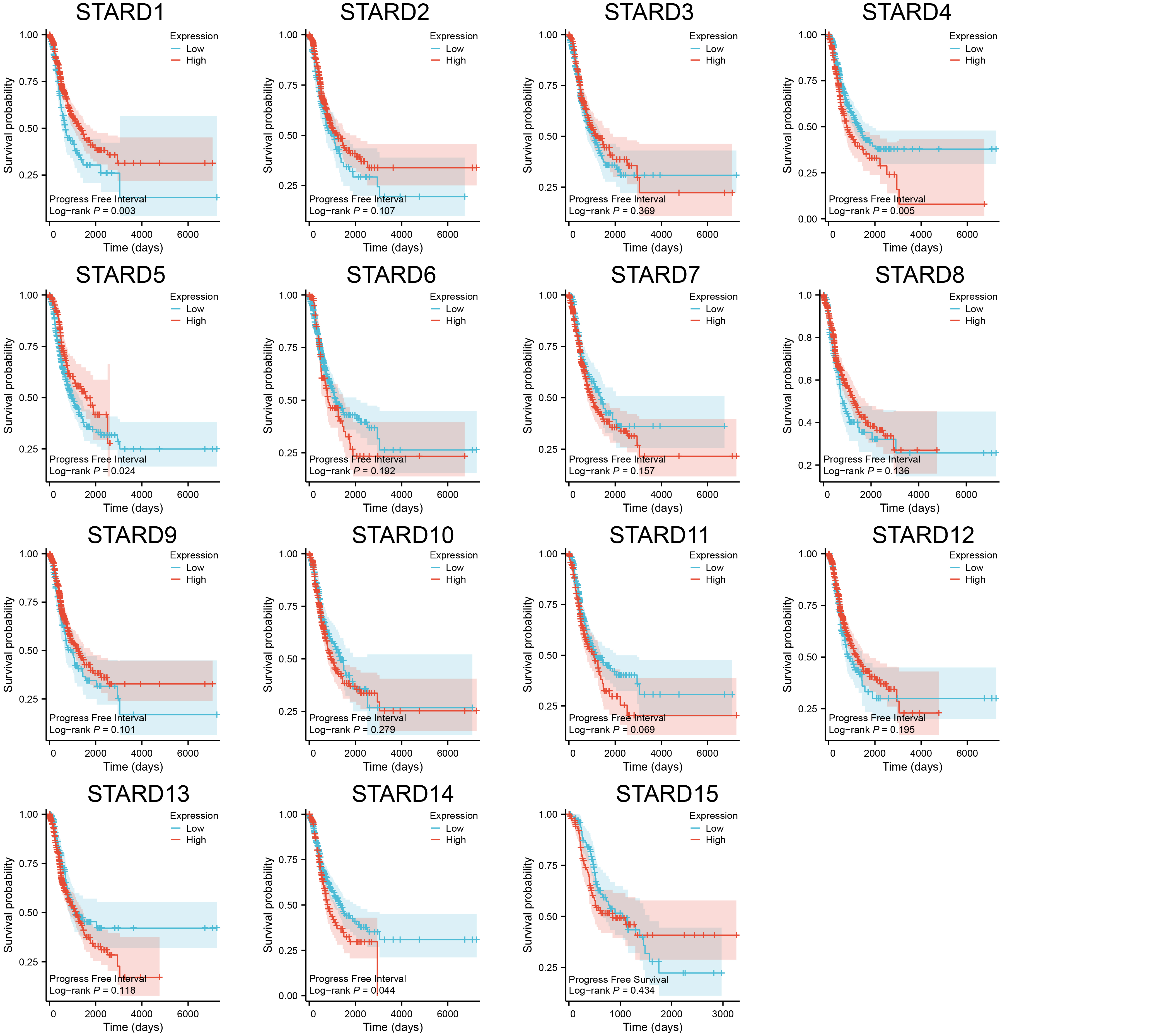

Supplement: Supplementary file 1 — Supplementary Table 1 Primer sequences of STARD12/14 and GAPDH; Supplementary Table 2 The significant differences of transcriptional expression of STARDs between diverse types of LUAD and normal samples (Oncomine); Supplementary Table 3 Univariate and multivariate Cox analysis of STARD12 and STARD14 in LUAD patients; Supplementary Table 4 Enrichment analysis results of STARDs in LUAD; Supplementary Figure 1 Pathological histological subgroup expression analysis of STARDs in LUAD; Supplementary Figure 2 Survival analysis on DFS of STARDs in LUAD; Supplementary Figure 3 Survival analysis on PFS of STARDs in LUAD; Supplementary Figure 4 Survival analysis on DSS of STARDs in LUAD. [file ijmsv20p1427s1.zip › Supplementary materials/Supplementary Figure 3.jpg]

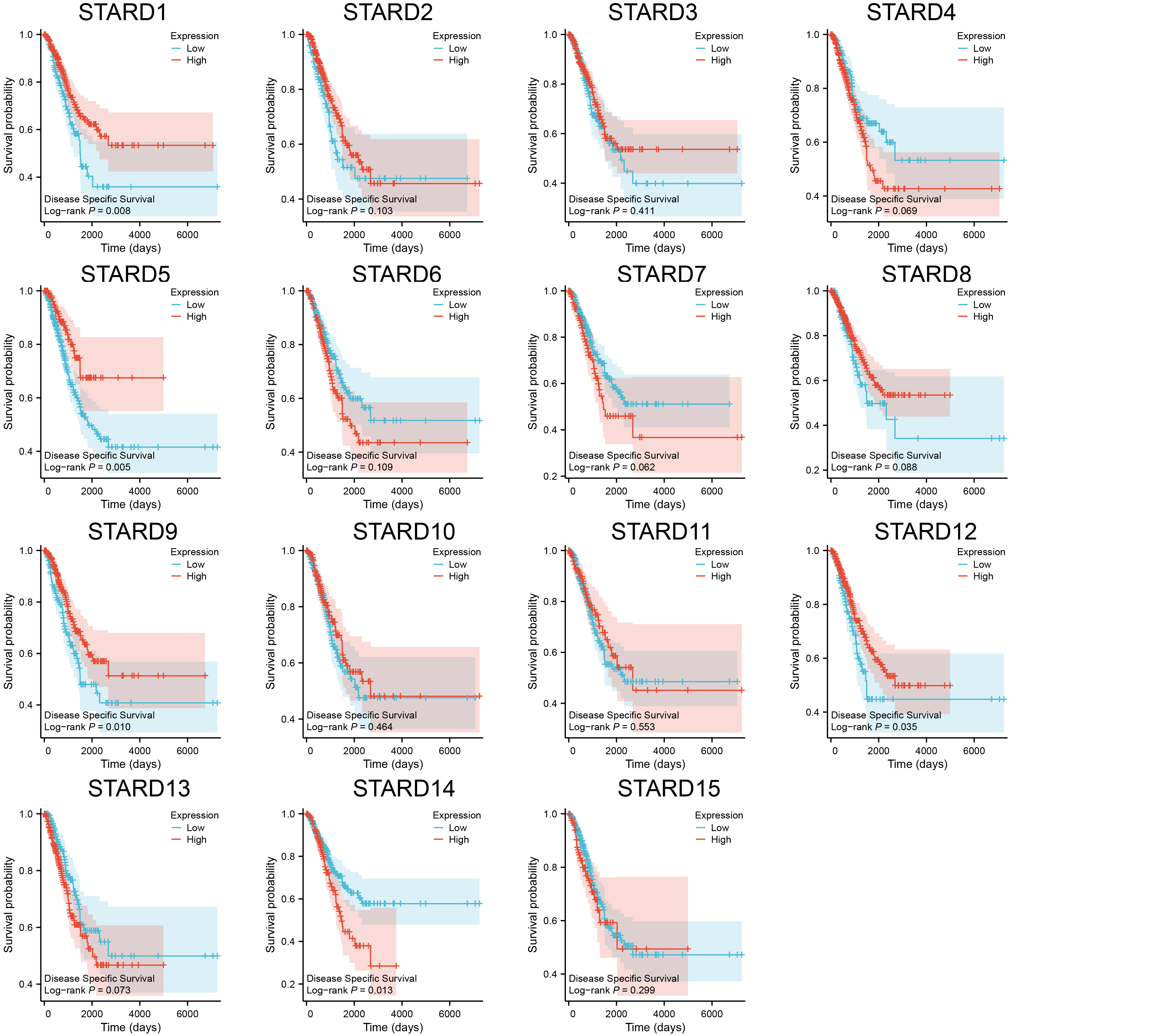

Supplement: Supplementary file 1 — Supplementary Table 1 Primer sequences of STARD12/14 and GAPDH; Supplementary Table 2 The significant differences of transcriptional expression of STARDs between diverse types of LUAD and normal samples (Oncomine); Supplementary Table 3 Univariate and multivariate Cox analysis of STARD12 and STARD14 in LUAD patients; Supplementary Table 4 Enrichment analysis results of STARDs in LUAD; Supplementary Figure 1 Pathological histological subgroup expression analysis of STARDs in LUAD; Supplementary Figure 2 Survival analysis on DFS of STARDs in LUAD; Supplementary Figure 3 Survival analysis on PFS of STARDs in LUAD; Supplementary Figure 4 Survival analysis on DSS of STARDs in LUAD. [file ijmsv20p1427s1.zip › Supplementary materials/Supplementary Figure 4.jpg]

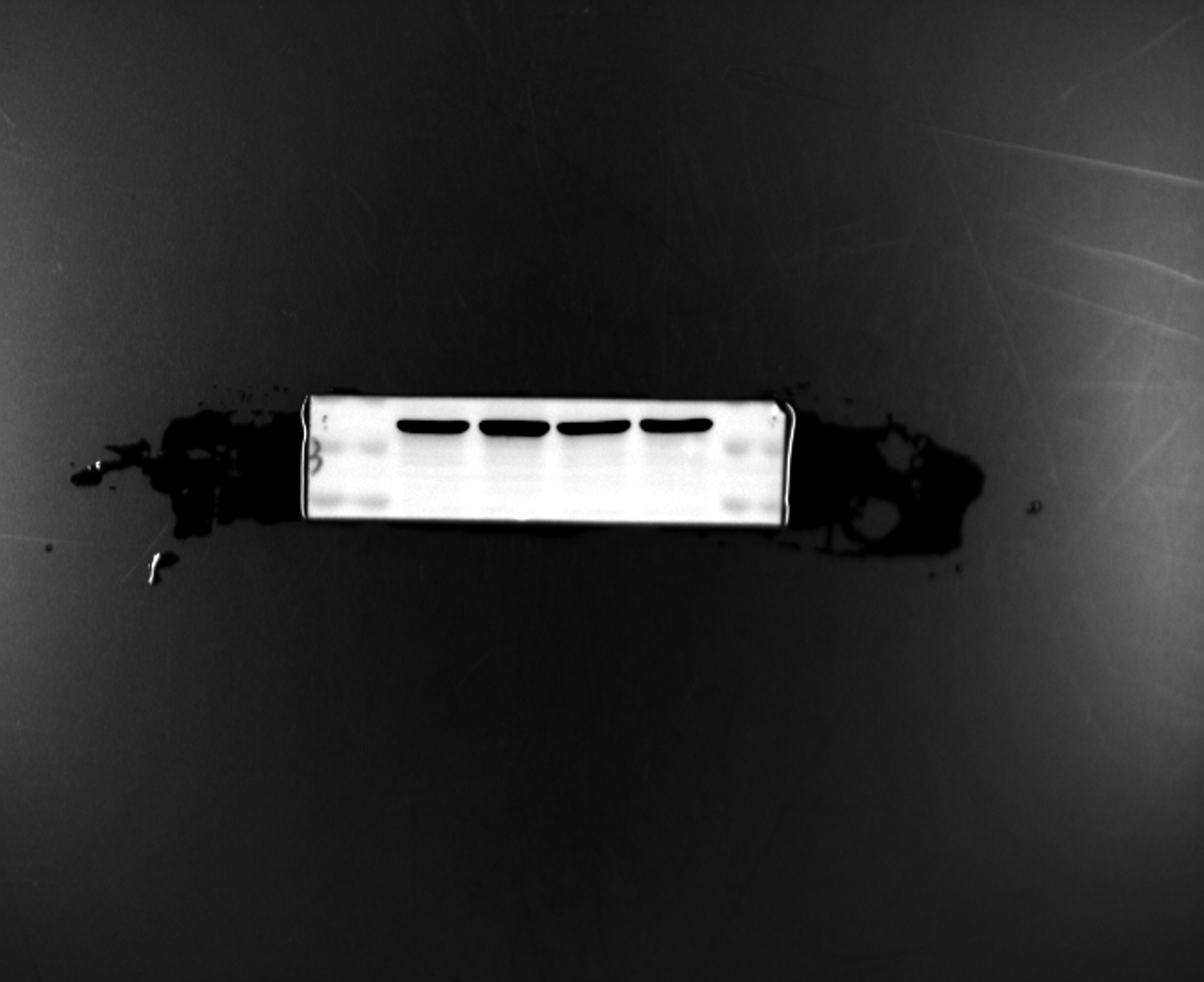

Supplement: Supplementary file 1 — Supplementary Table 1 Primer sequences of STARD12/14 and GAPDH; Supplementary Table 2 The significant differences of transcriptional expression of STARDs between diverse types of LUAD and normal samples (Oncomine); Supplementary Table 3 Univariate and multivariate Cox analysis of STARD12 and STARD14 in LUAD patients; Supplementary Table 4 Enrichment analysis results of STARDs in LUAD; Supplementary Figure 1 Pathological histological subgroup expression analysis of STARDs in LUAD; Supplementary Figure 2 Survival analysis on DFS of STARDs in LUAD; Supplementary Figure 3 Survival analysis on PFS of STARDs in LUAD; Supplementary Figure 4 Survival analysis on DSS of STARDs in LUAD. [file ijmsv20p1427s1.zip › Supplementary materials/Western blot-Raw data/STARD12/GADPH-R.Tif]

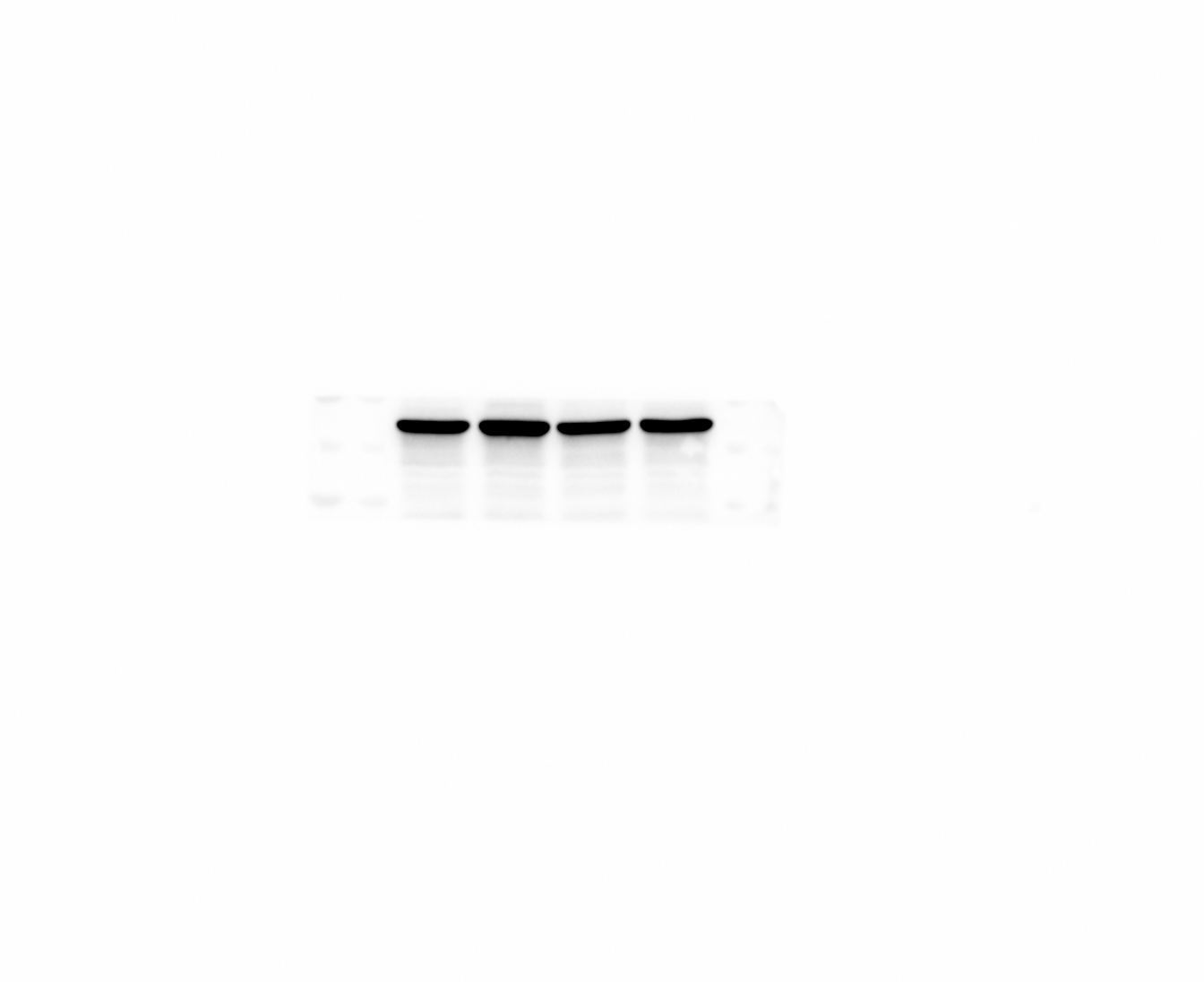

Supplement: Supplementary file 1 — Supplementary Table 1 Primer sequences of STARD12/14 and GAPDH; Supplementary Table 2 The significant differences of transcriptional expression of STARDs between diverse types of LUAD and normal samples (Oncomine); Supplementary Table 3 Univariate and multivariate Cox analysis of STARD12 and STARD14 in LUAD patients; Supplementary Table 4 Enrichment analysis results of STARDs in LUAD; Supplementary Figure 1 Pathological histological subgroup expression analysis of STARDs in LUAD; Supplementary Figure 2 Survival analysis on DFS of STARDs in LUAD; Supplementary Figure 3 Survival analysis on PFS of STARDs in LUAD; Supplementary Figure 4 Survival analysis on DSS of STARDs in LUAD. [file ijmsv20p1427s1.zip › Supplementary materials/Western blot-Raw data/STARD12/GADPH.Tif]

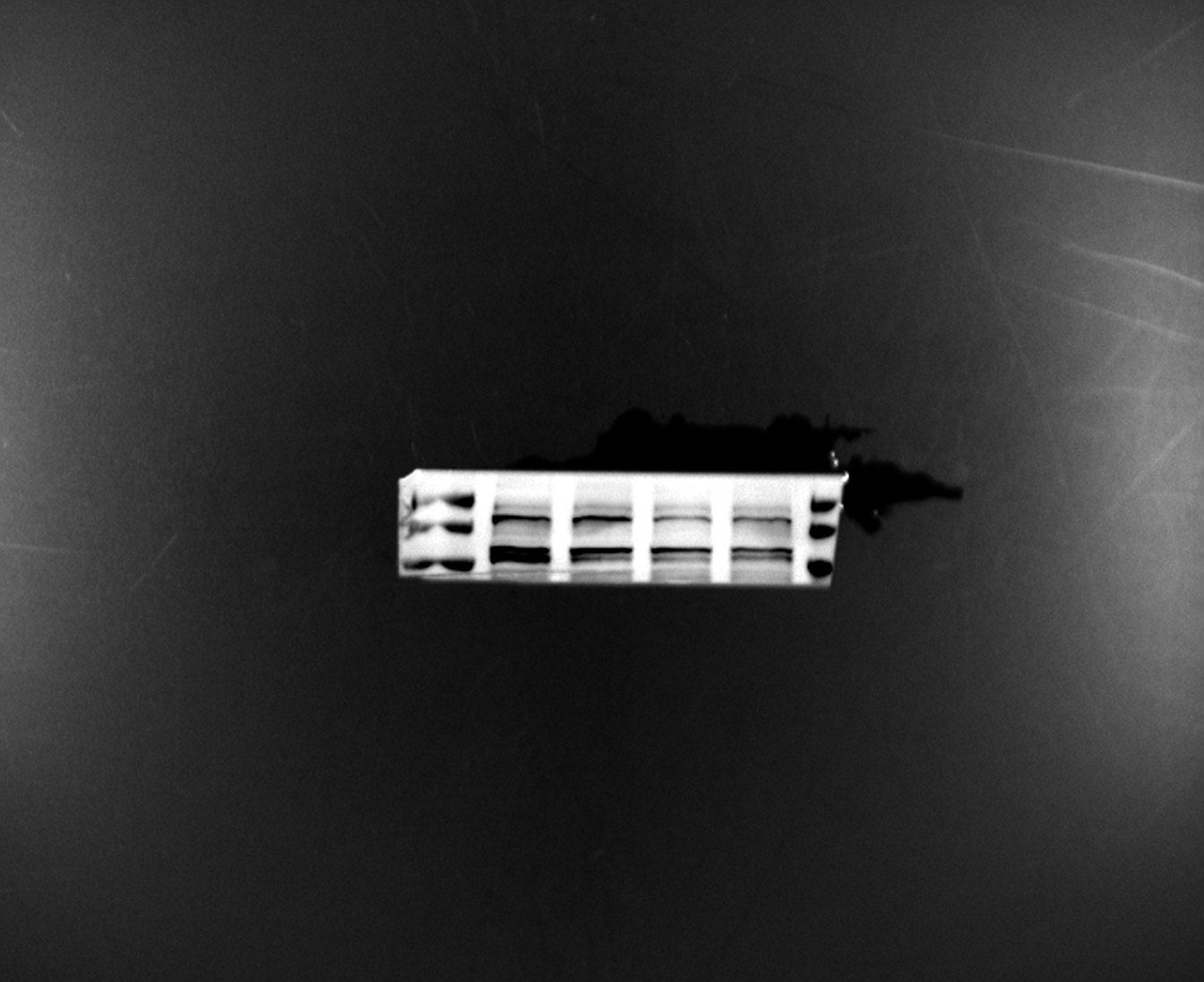

Supplement: Supplementary file 1 — Supplementary Table 1 Primer sequences of STARD12/14 and GAPDH; Supplementary Table 2 The significant differences of transcriptional expression of STARDs between diverse types of LUAD and normal samples (Oncomine); Supplementary Table 3 Univariate and multivariate Cox analysis of STARD12 and STARD14 in LUAD patients; Supplementary Table 4 Enrichment analysis results of STARDs in LUAD; Supplementary Figure 1 Pathological histological subgroup expression analysis of STARDs in LUAD; Supplementary Figure 2 Survival analysis on DFS of STARDs in LUAD; Supplementary Figure 3 Survival analysis on PFS of STARDs in LUAD; Supplementary Figure 4 Survival analysis on DSS of STARDs in LUAD. [file ijmsv20p1427s1.zip › Supplementary materials/Western blot-Raw data/STARD12/STARD12-R.Tif]

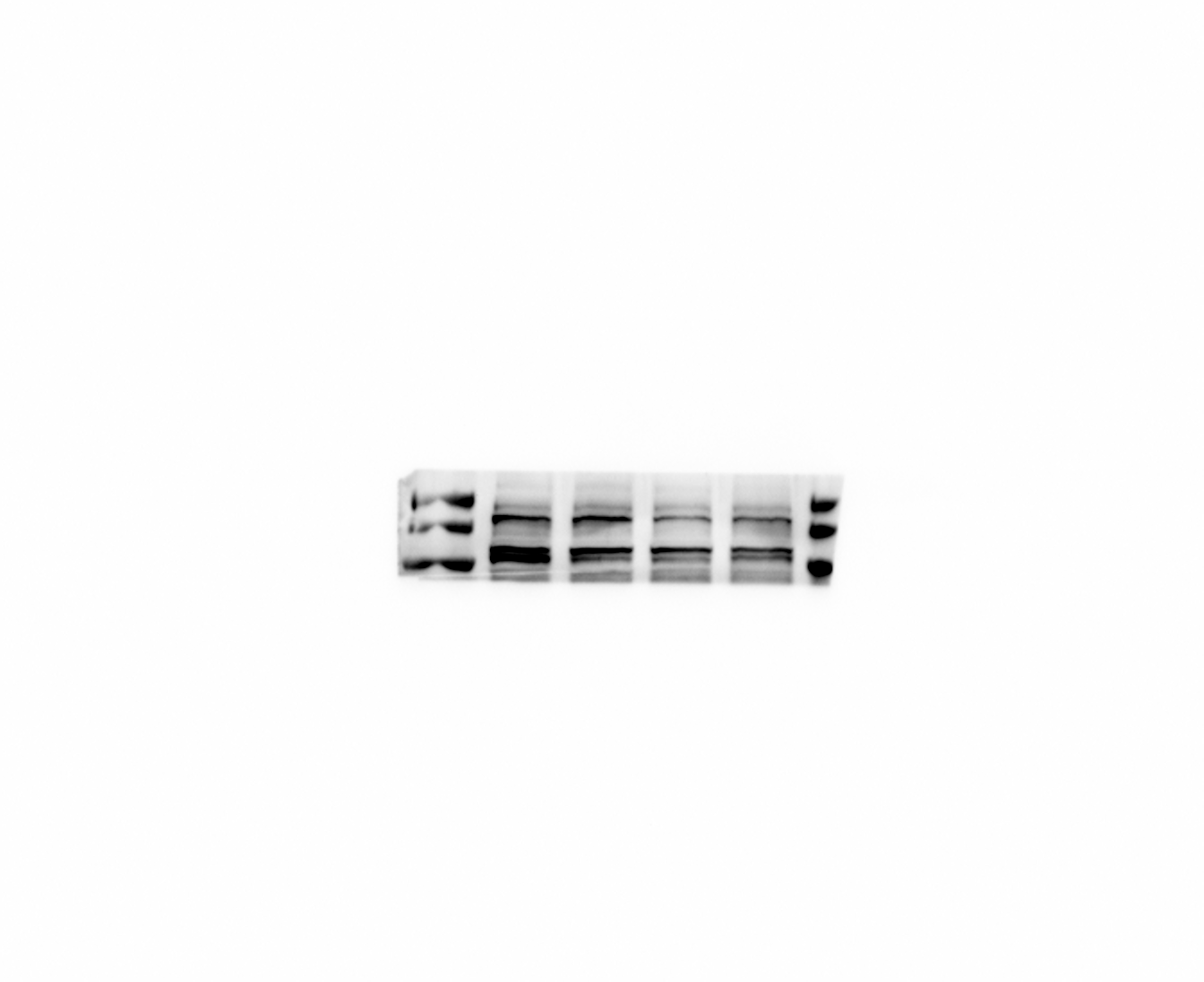

Supplement: Supplementary file 1 — Supplementary Table 1 Primer sequences of STARD12/14 and GAPDH; Supplementary Table 2 The significant differences of transcriptional expression of STARDs between diverse types of LUAD and normal samples (Oncomine); Supplementary Table 3 Univariate and multivariate Cox analysis of STARD12 and STARD14 in LUAD patients; Supplementary Table 4 Enrichment analysis results of STARDs in LUAD; Supplementary Figure 1 Pathological histological subgroup expression analysis of STARDs in LUAD; Supplementary Figure 2 Survival analysis on DFS of STARDs in LUAD; Supplementary Figure 3 Survival analysis on PFS of STARDs in LUAD; Supplementary Figure 4 Survival analysis on DSS of STARDs in LUAD. [file ijmsv20p1427s1.zip › Supplementary materials/Western blot-Raw data/STARD12/STARD12.Tif]

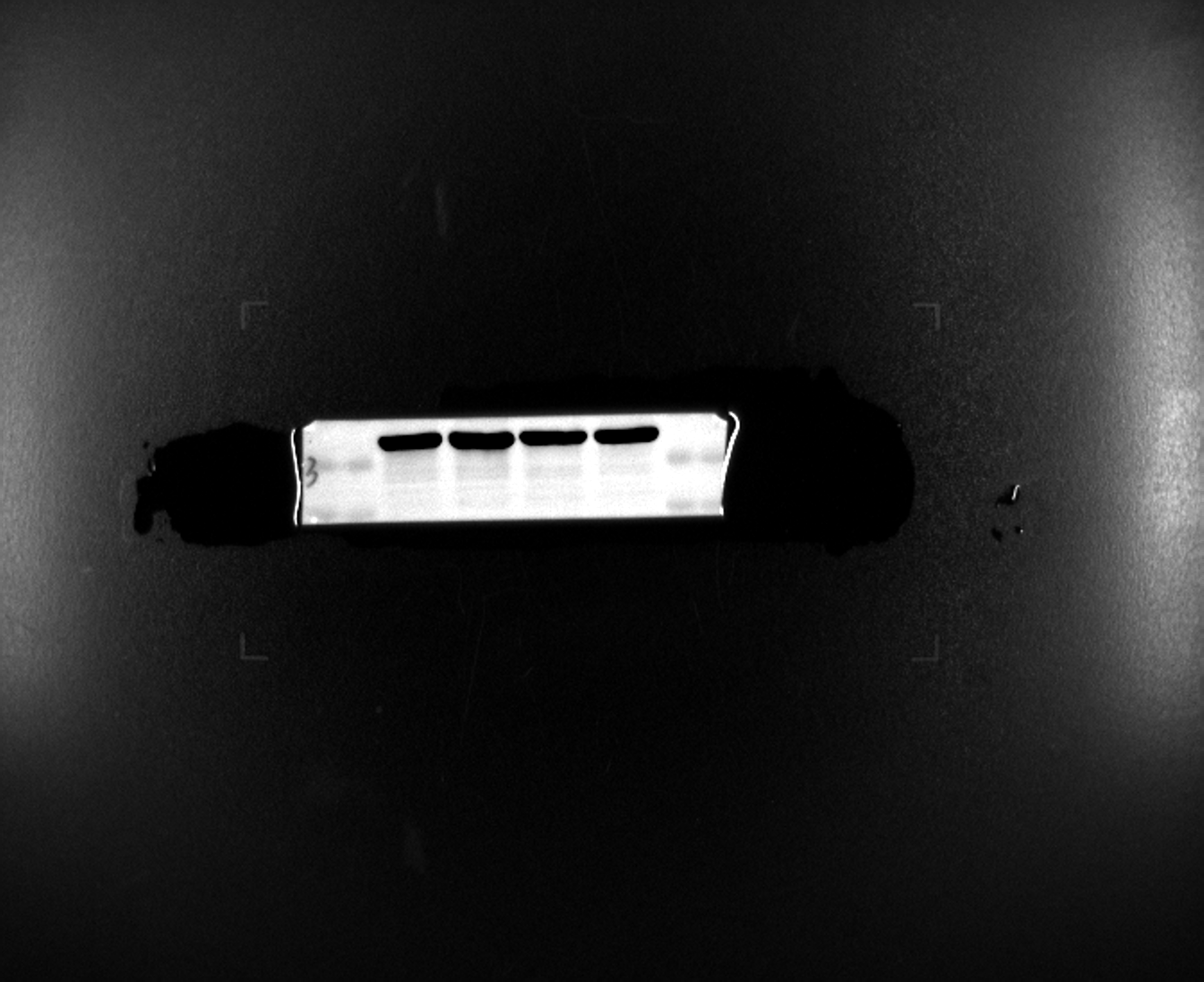

Supplement: Supplementary file 1 — Supplementary Table 1 Primer sequences of STARD12/14 and GAPDH; Supplementary Table 2 The significant differences of transcriptional expression of STARDs between diverse types of LUAD and normal samples (Oncomine); Supplementary Table 3 Univariate and multivariate Cox analysis of STARD12 and STARD14 in LUAD patients; Supplementary Table 4 Enrichment analysis results of STARDs in LUAD; Supplementary Figure 1 Pathological histological subgroup expression analysis of STARDs in LUAD; Supplementary Figure 2 Survival analysis on DFS of STARDs in LUAD; Supplementary Figure 3 Survival analysis on PFS of STARDs in LUAD; Supplementary Figure 4 Survival analysis on DSS of STARDs in LUAD. [file ijmsv20p1427s1.zip › Supplementary materials/Western blot-Raw data/STARD14/GADPH-R.Tif]

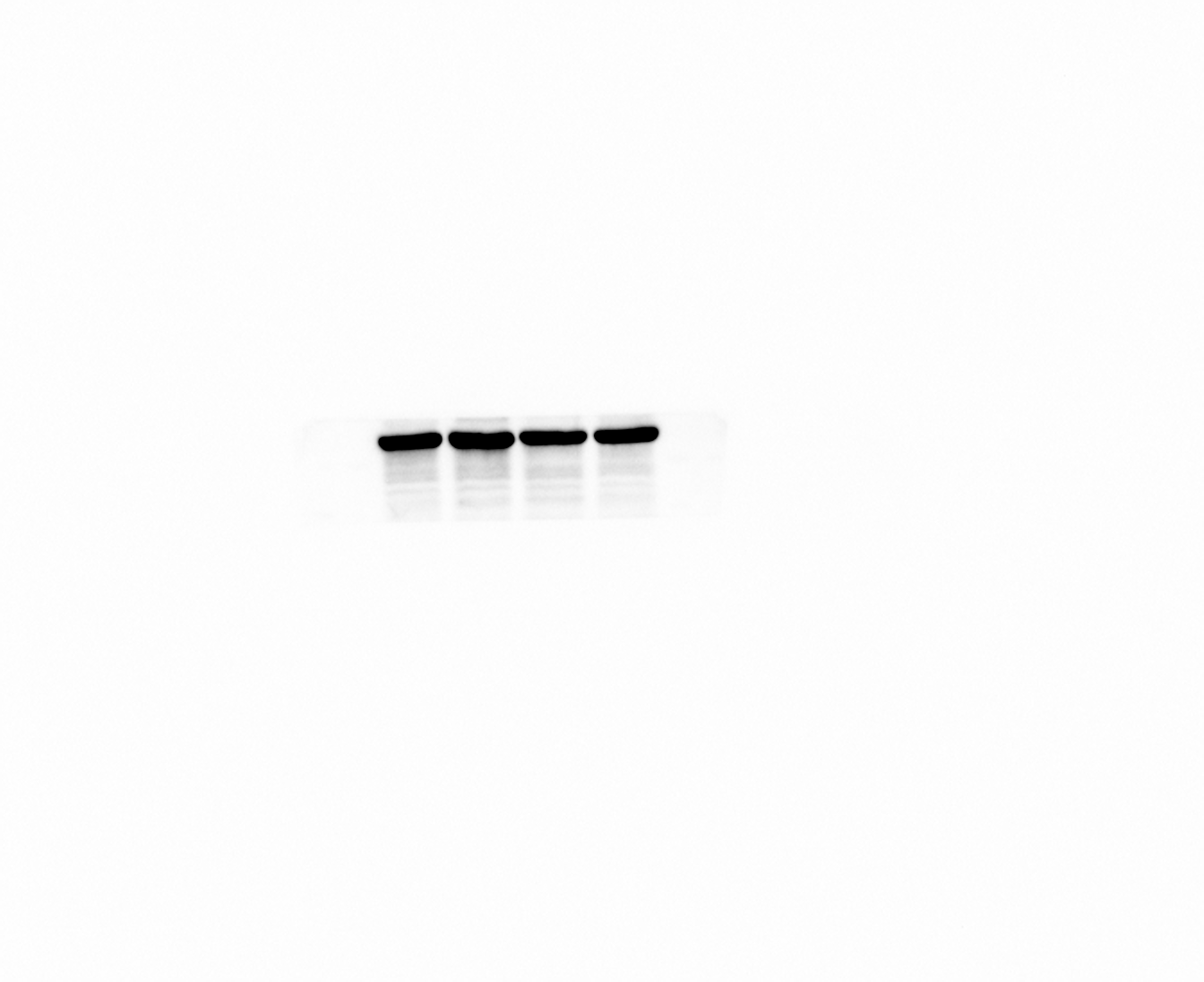

Supplement: Supplementary file 1 — Supplementary Table 1 Primer sequences of STARD12/14 and GAPDH; Supplementary Table 2 The significant differences of transcriptional expression of STARDs between diverse types of LUAD and normal samples (Oncomine); Supplementary Table 3 Univariate and multivariate Cox analysis of STARD12 and STARD14 in LUAD patients; Supplementary Table 4 Enrichment analysis results of STARDs in LUAD; Supplementary Figure 1 Pathological histological subgroup expression analysis of STARDs in LUAD; Supplementary Figure 2 Survival analysis on DFS of STARDs in LUAD; Supplementary Figure 3 Survival analysis on PFS of STARDs in LUAD; Supplementary Figure 4 Survival analysis on DSS of STARDs in LUAD. [file ijmsv20p1427s1.zip › Supplementary materials/Western blot-Raw data/STARD14/GADPH.Tif]

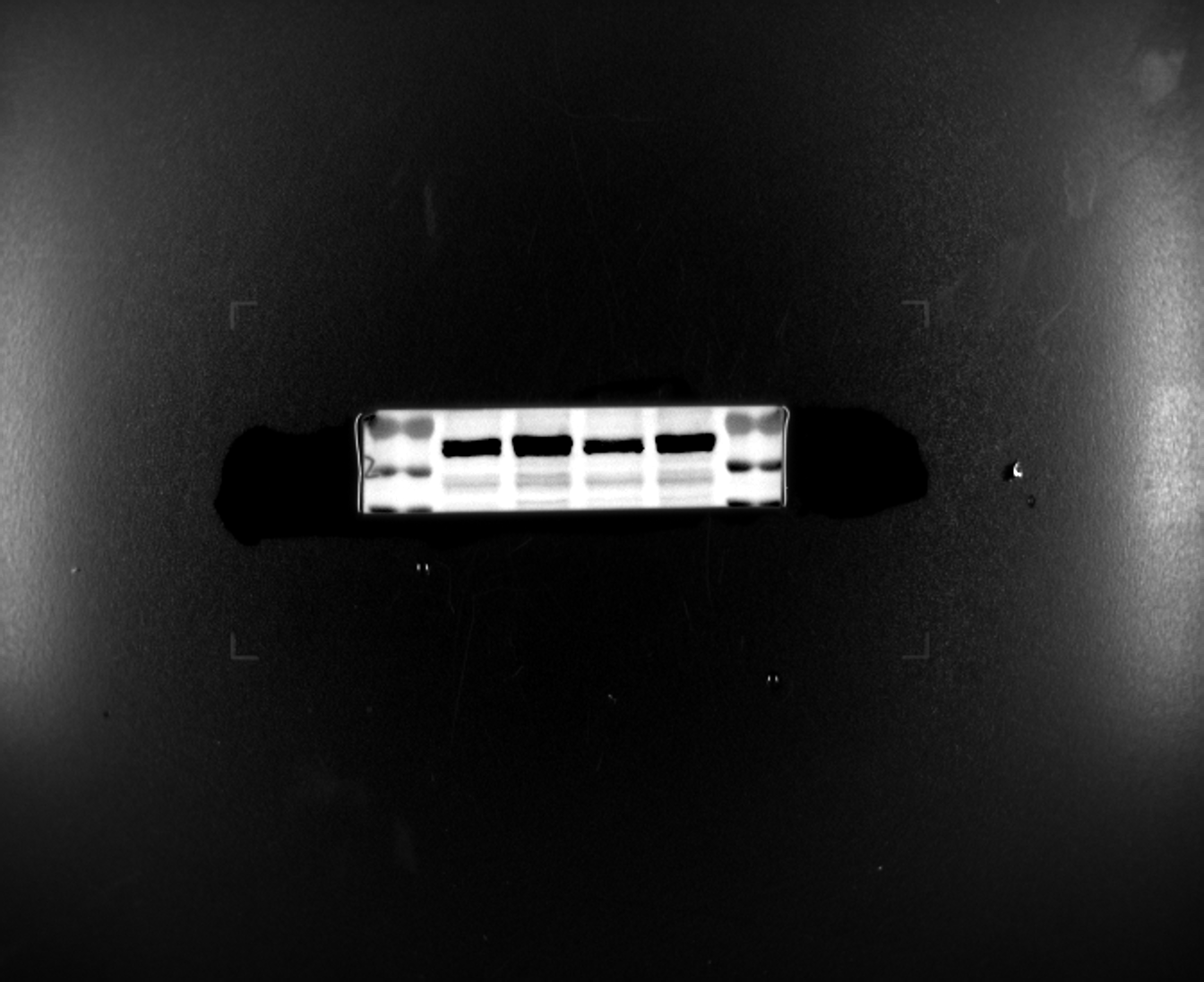

Supplement: Supplementary file 1 — Supplementary Table 1 Primer sequences of STARD12/14 and GAPDH; Supplementary Table 2 The significant differences of transcriptional expression of STARDs between diverse types of LUAD and normal samples (Oncomine); Supplementary Table 3 Univariate and multivariate Cox analysis of STARD12 and STARD14 in LUAD patients; Supplementary Table 4 Enrichment analysis results of STARDs in LUAD; Supplementary Figure 1 Pathological histological subgroup expression analysis of STARDs in LUAD; Supplementary Figure 2 Survival analysis on DFS of STARDs in LUAD; Supplementary Figure 3 Survival analysis on PFS of STARDs in LUAD; Supplementary Figure 4 Survival analysis on DSS of STARDs in LUAD. [file ijmsv20p1427s1.zip › Supplementary materials/Western blot-Raw data/STARD14/STARD14-R.Tif]

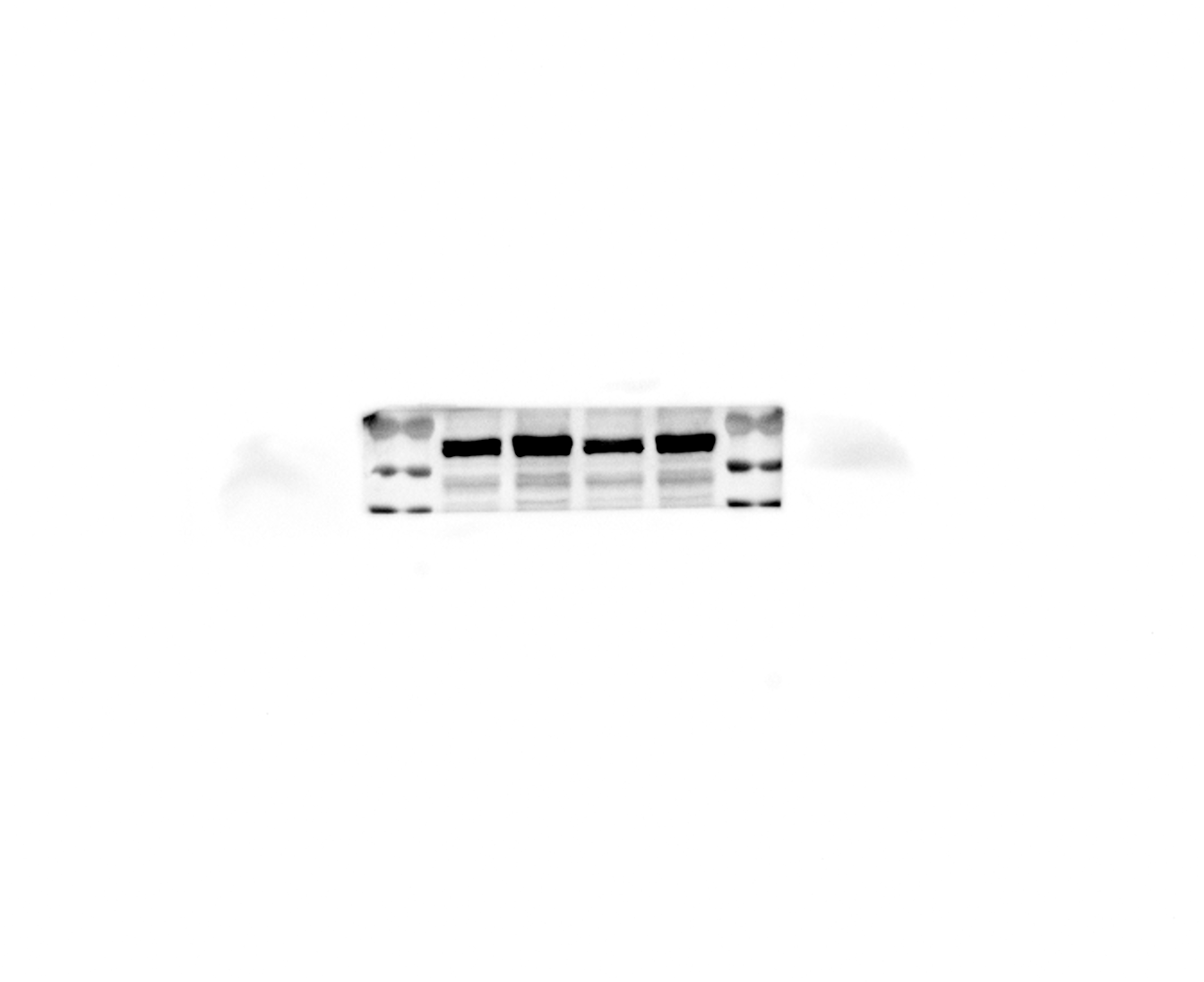

Supplement: Supplementary file 1 — Supplementary Table 1 Primer sequences of STARD12/14 and GAPDH; Supplementary Table 2 The significant differences of transcriptional expression of STARDs between diverse types of LUAD and normal samples (Oncomine); Supplementary Table 3 Univariate and multivariate Cox analysis of STARD12 and STARD14 in LUAD patients; Supplementary Table 4 Enrichment analysis results of STARDs in LUAD; Supplementary Figure 1 Pathological histological subgroup expression analysis of STARDs in LUAD; Supplementary Figure 2 Survival analysis on DFS of STARDs in LUAD; Supplementary Figure 3 Survival analysis on PFS of STARDs in LUAD; Supplementary Figure 4 Survival analysis on DSS of STARDs in LUAD. [file ijmsv20p1427s1.zip › Supplementary materials/Western blot-Raw data/STARD14/STARD14.Tif]

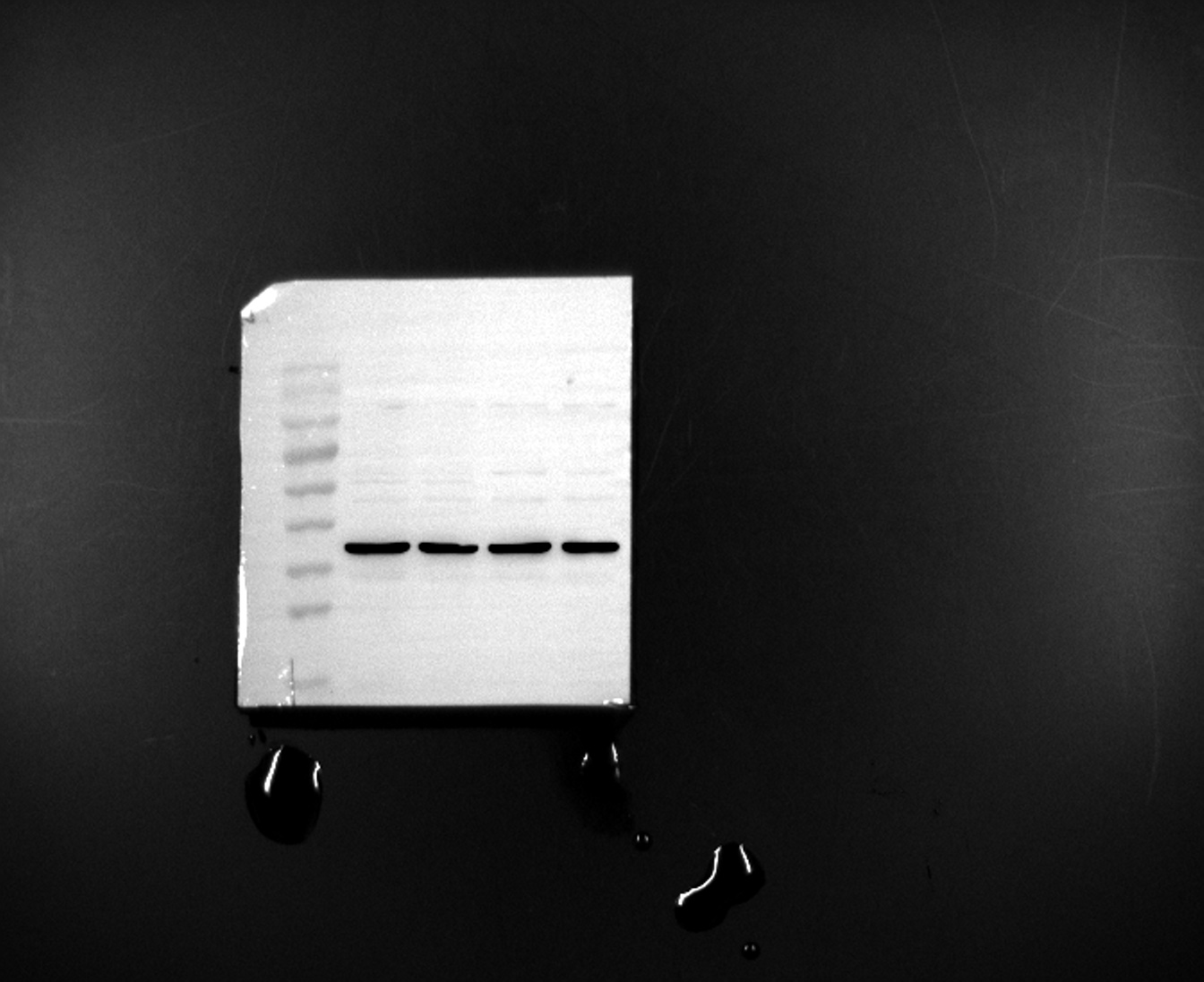

Supplement: Supplementary file 1 — Supplementary Table 1 Primer sequences of STARD12/14 and GAPDH; Supplementary Table 2 The significant differences of transcriptional expression of STARDs between diverse types of LUAD and normal samples (Oncomine); Supplementary Table 3 Univariate and multivariate Cox analysis of STARD12 and STARD14 in LUAD patients; Supplementary Table 4 Enrichment analysis results of STARDs in LUAD; Supplementary Figure 1 Pathological histological subgroup expression analysis of STARDs in LUAD; Supplementary Figure 2 Survival analysis on DFS of STARDs in LUAD; Supplementary Figure 3 Survival analysis on PFS of STARDs in LUAD; Supplementary Figure 4 Survival analysis on DSS of STARDs in LUAD. [file ijmsv20p1427s1.zip › Supplementary materials/Western blot-Raw data/whole gel/STARD12/GAPDH-R.Tif]

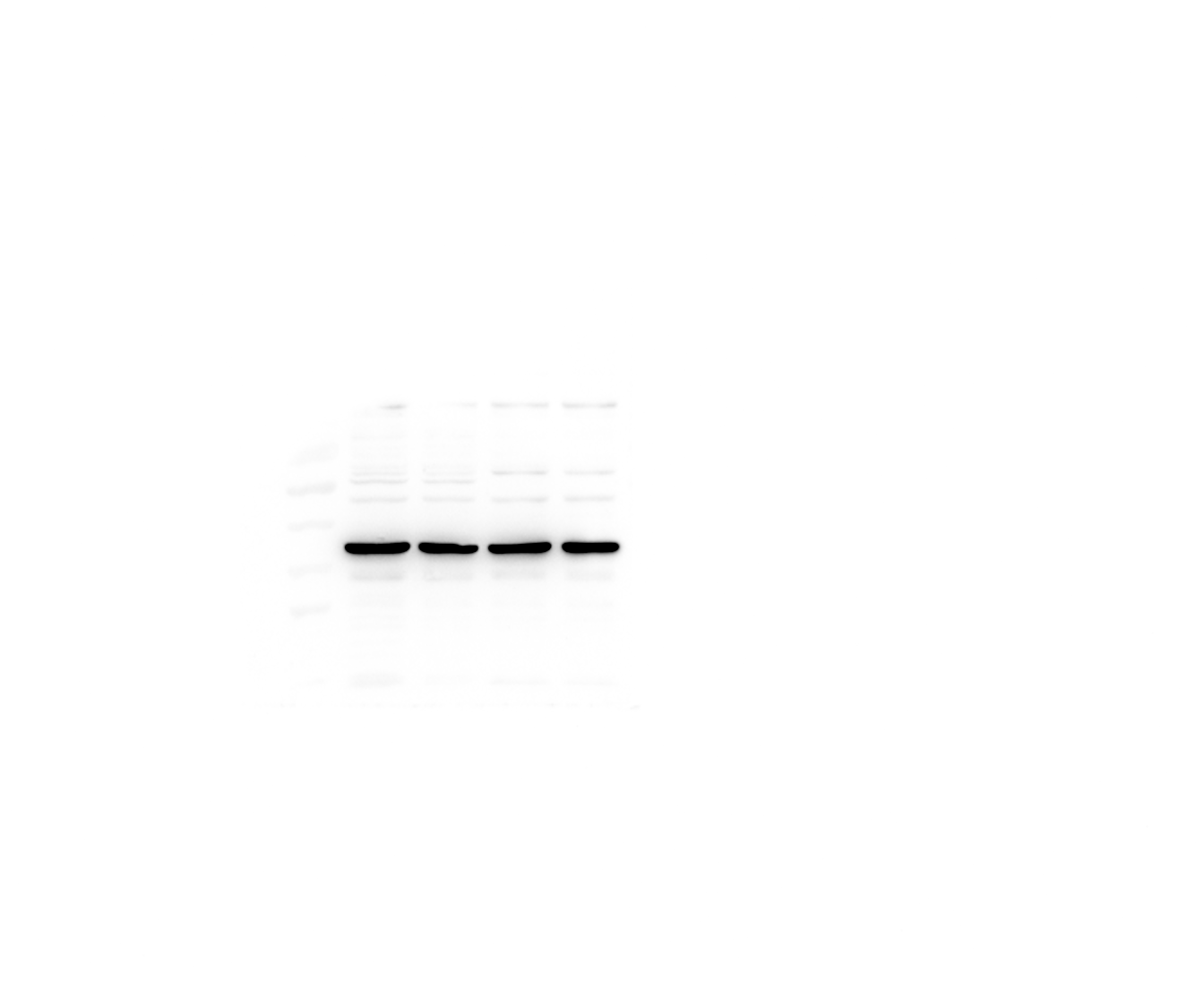

Supplement: Supplementary file 1 — Supplementary Table 1 Primer sequences of STARD12/14 and GAPDH; Supplementary Table 2 The significant differences of transcriptional expression of STARDs between diverse types of LUAD and normal samples (Oncomine); Supplementary Table 3 Univariate and multivariate Cox analysis of STARD12 and STARD14 in LUAD patients; Supplementary Table 4 Enrichment analysis results of STARDs in LUAD; Supplementary Figure 1 Pathological histological subgroup expression analysis of STARDs in LUAD; Supplementary Figure 2 Survival analysis on DFS of STARDs in LUAD; Supplementary Figure 3 Survival analysis on PFS of STARDs in LUAD; Supplementary Figure 4 Survival analysis on DSS of STARDs in LUAD. [file ijmsv20p1427s1.zip › Supplementary materials/Western blot-Raw data/whole gel/STARD12/GAPDH.Tif]

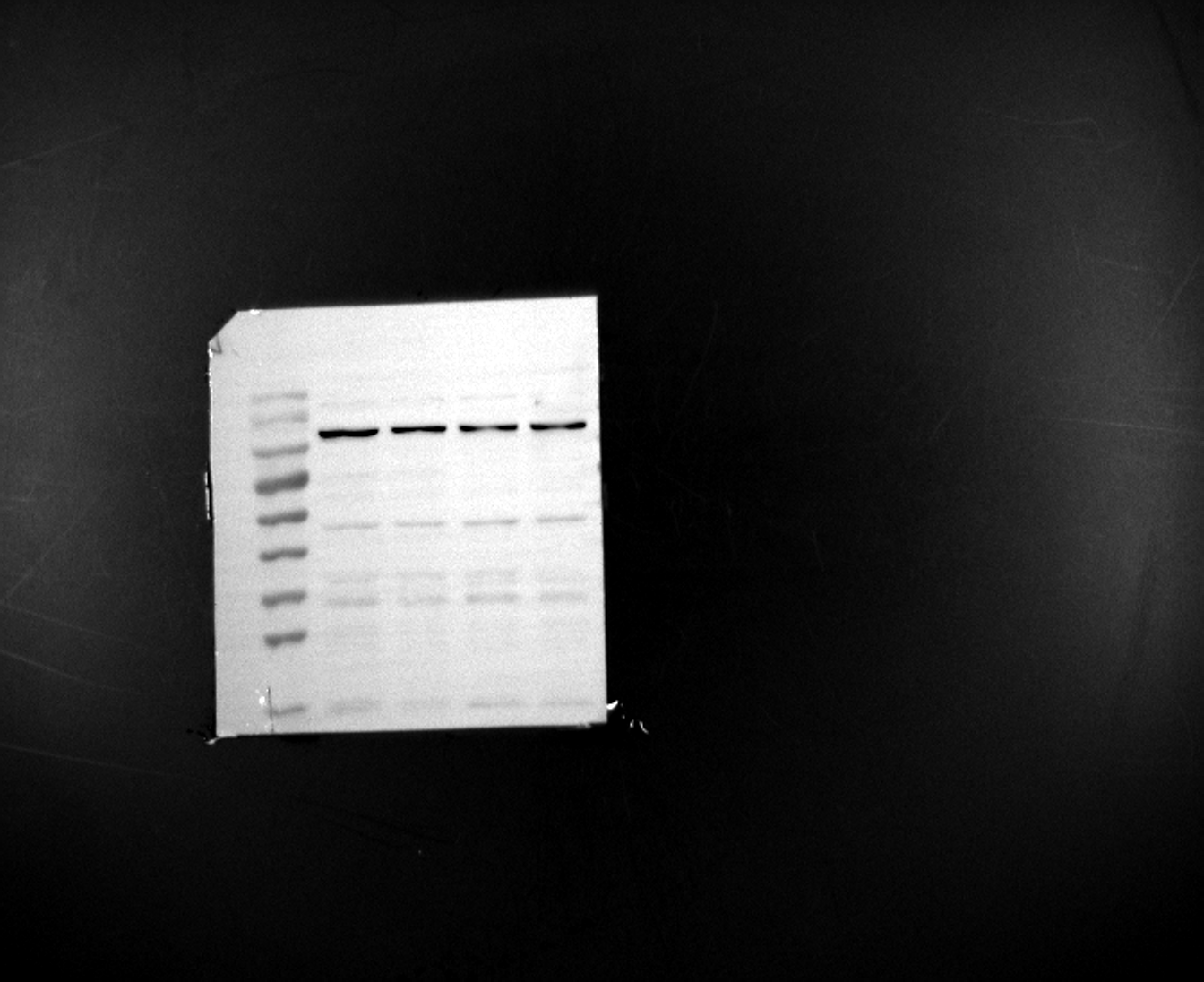

Supplement: Supplementary file 1 — Supplementary Table 1 Primer sequences of STARD12/14 and GAPDH; Supplementary Table 2 The significant differences of transcriptional expression of STARDs between diverse types of LUAD and normal samples (Oncomine); Supplementary Table 3 Univariate and multivariate Cox analysis of STARD12 and STARD14 in LUAD patients; Supplementary Table 4 Enrichment analysis results of STARDs in LUAD; Supplementary Figure 1 Pathological histological subgroup expression analysis of STARDs in LUAD; Supplementary Figure 2 Survival analysis on DFS of STARDs in LUAD; Supplementary Figure 3 Survival analysis on PFS of STARDs in LUAD; Supplementary Figure 4 Survival analysis on DSS of STARDs in LUAD. [file ijmsv20p1427s1.zip › Supplementary materials/Western blot-Raw data/whole gel/STARD12/STARD12-R.Tif]

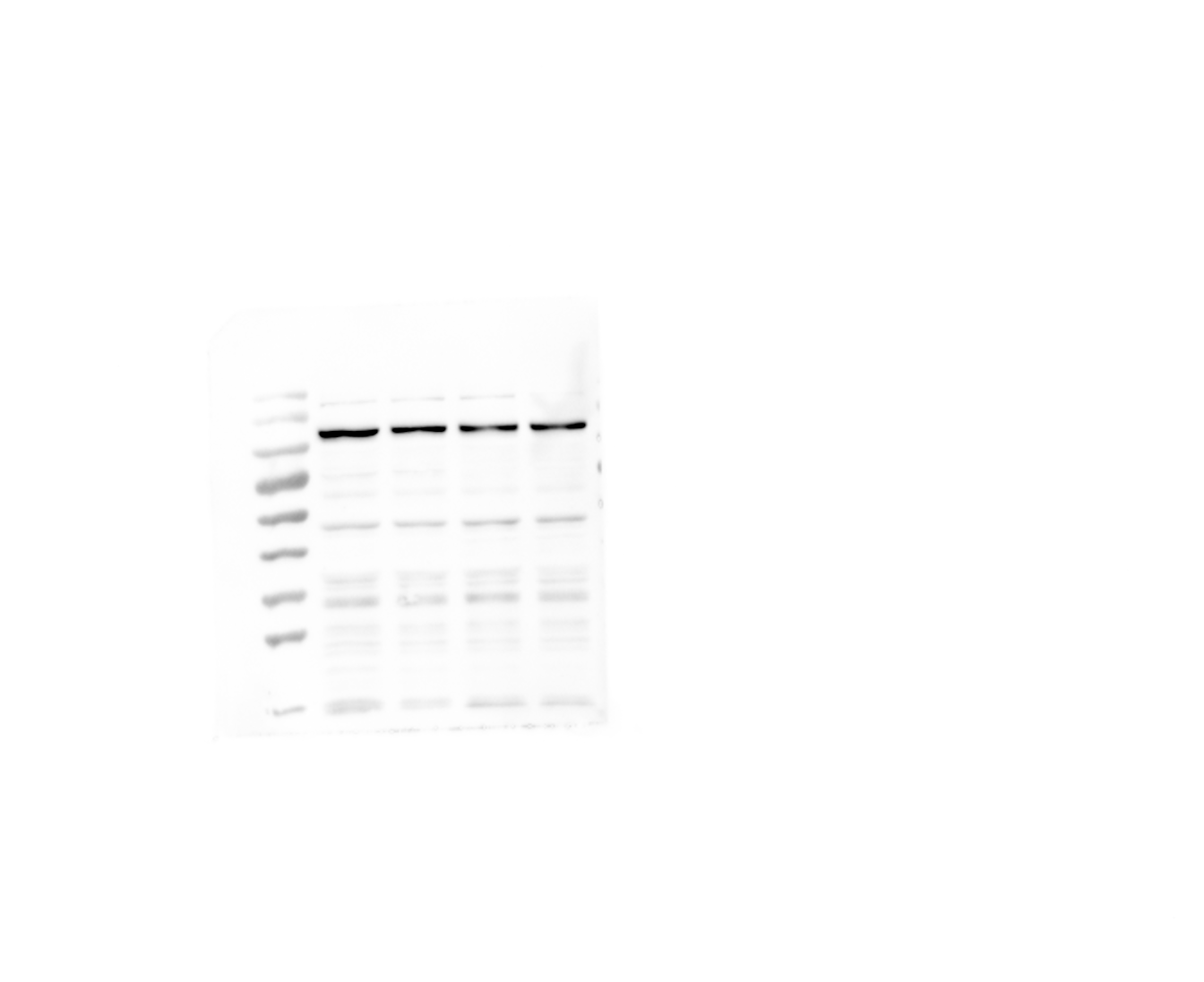

Supplement: Supplementary file 1 — Supplementary Table 1 Primer sequences of STARD12/14 and GAPDH; Supplementary Table 2 The significant differences of transcriptional expression of STARDs between diverse types of LUAD and normal samples (Oncomine); Supplementary Table 3 Univariate and multivariate Cox analysis of STARD12 and STARD14 in LUAD patients; Supplementary Table 4 Enrichment analysis results of STARDs in LUAD; Supplementary Figure 1 Pathological histological subgroup expression analysis of STARDs in LUAD; Supplementary Figure 2 Survival analysis on DFS of STARDs in LUAD; Supplementary Figure 3 Survival analysis on PFS of STARDs in LUAD; Supplementary Figure 4 Survival analysis on DSS of STARDs in LUAD. [file ijmsv20p1427s1.zip › Supplementary materials/Western blot-Raw data/whole gel/STARD12/STARD12.Tif]

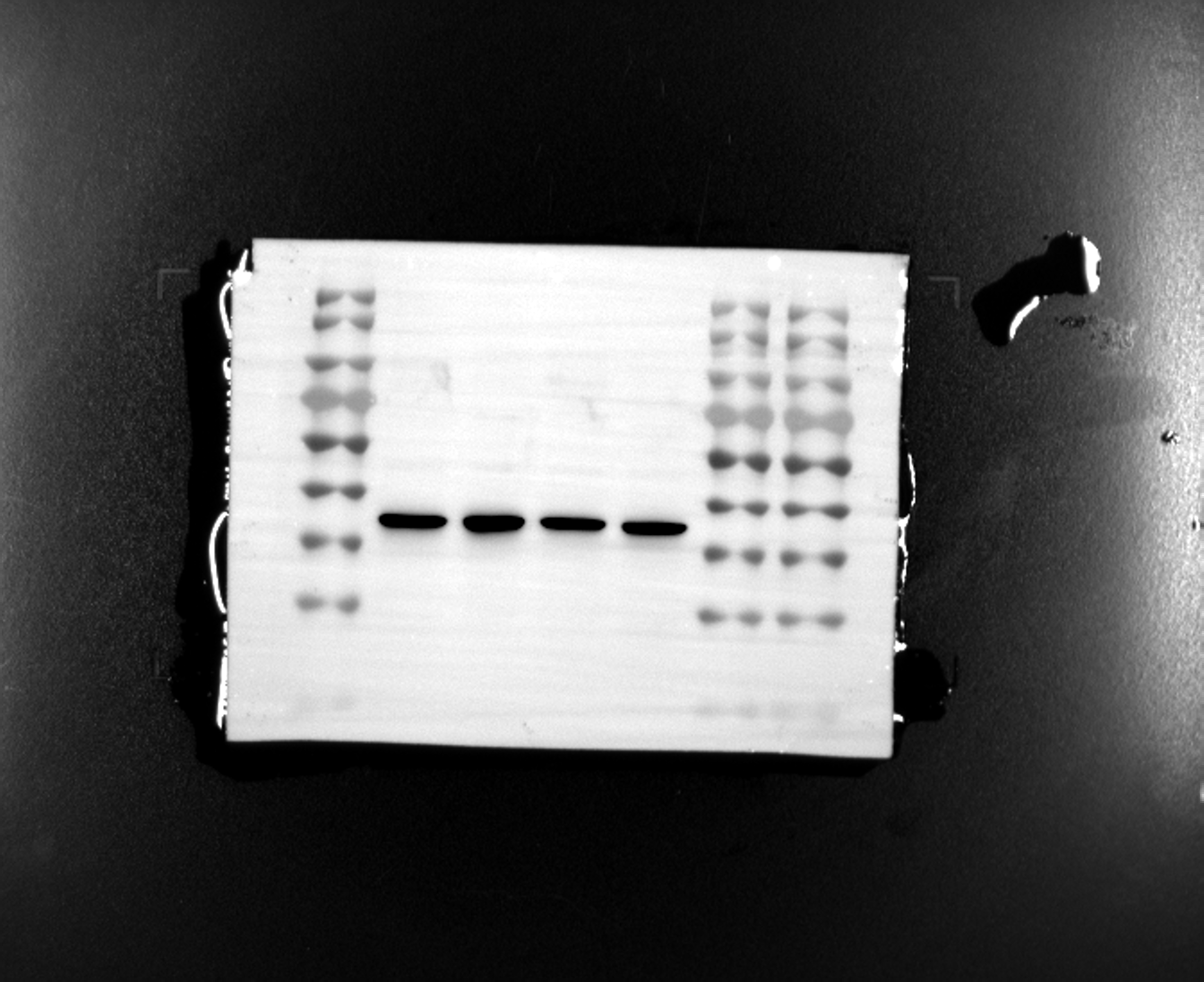

Supplement: Supplementary file 1 — Supplementary Table 1 Primer sequences of STARD12/14 and GAPDH; Supplementary Table 2 The significant differences of transcriptional expression of STARDs between diverse types of LUAD and normal samples (Oncomine); Supplementary Table 3 Univariate and multivariate Cox analysis of STARD12 and STARD14 in LUAD patients; Supplementary Table 4 Enrichment analysis results of STARDs in LUAD; Supplementary Figure 1 Pathological histological subgroup expression analysis of STARDs in LUAD; Supplementary Figure 2 Survival analysis on DFS of STARDs in LUAD; Supplementary Figure 3 Survival analysis on PFS of STARDs in LUAD; Supplementary Figure 4 Survival analysis on DSS of STARDs in LUAD. [file ijmsv20p1427s1.zip › Supplementary materials/Western blot-Raw data/whole gel/STARD14/GAPDH-R.Tif]

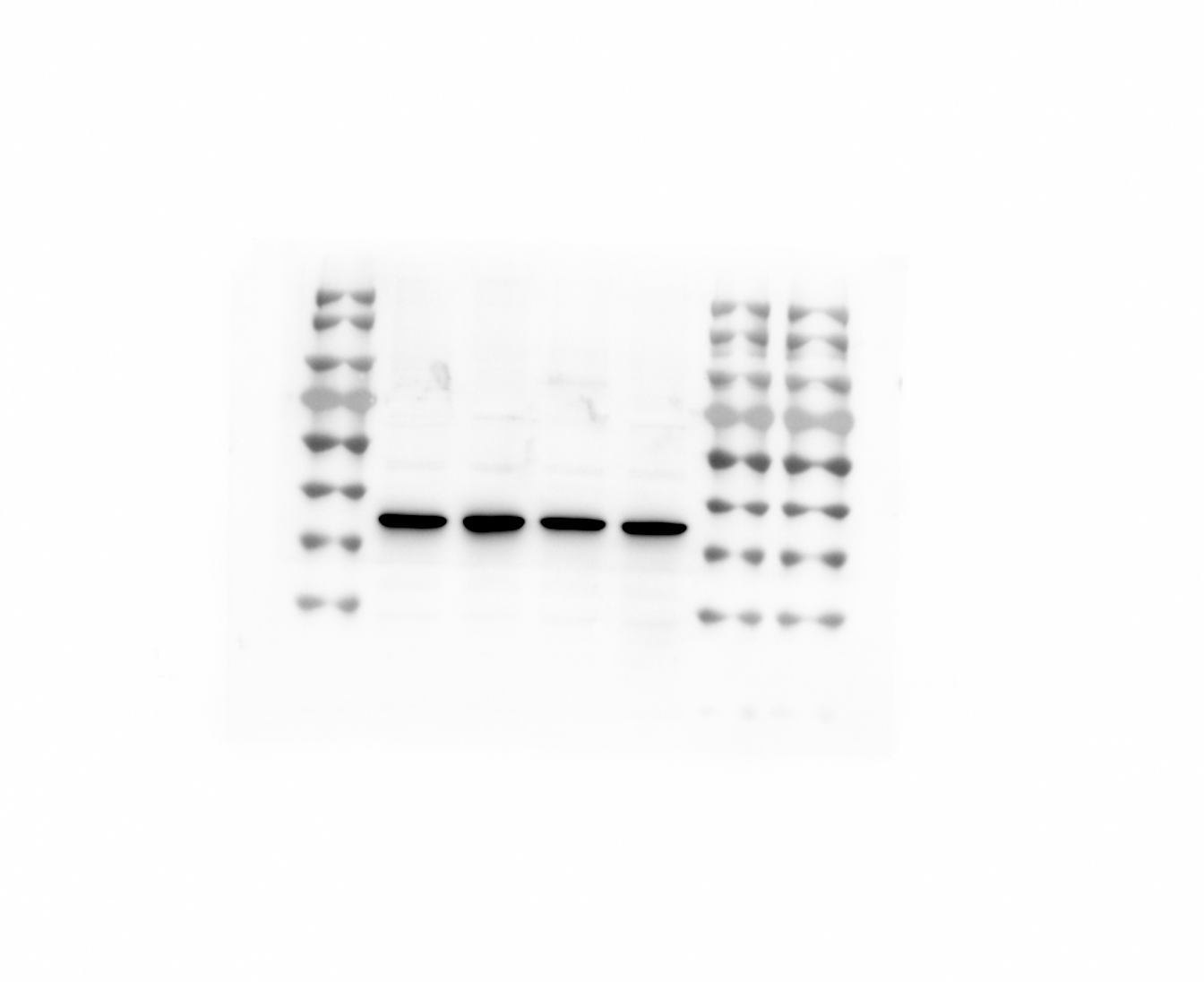

Supplement: Supplementary file 1 — Supplementary Table 1 Primer sequences of STARD12/14 and GAPDH; Supplementary Table 2 The significant differences of transcriptional expression of STARDs between diverse types of LUAD and normal samples (Oncomine); Supplementary Table 3 Univariate and multivariate Cox analysis of STARD12 and STARD14 in LUAD patients; Supplementary Table 4 Enrichment analysis results of STARDs in LUAD; Supplementary Figure 1 Pathological histological subgroup expression analysis of STARDs in LUAD; Supplementary Figure 2 Survival analysis on DFS of STARDs in LUAD; Supplementary Figure 3 Survival analysis on PFS of STARDs in LUAD; Supplementary Figure 4 Survival analysis on DSS of STARDs in LUAD. [file ijmsv20p1427s1.zip › Supplementary materials/Western blot-Raw data/whole gel/STARD14/GAPDH.Tif]

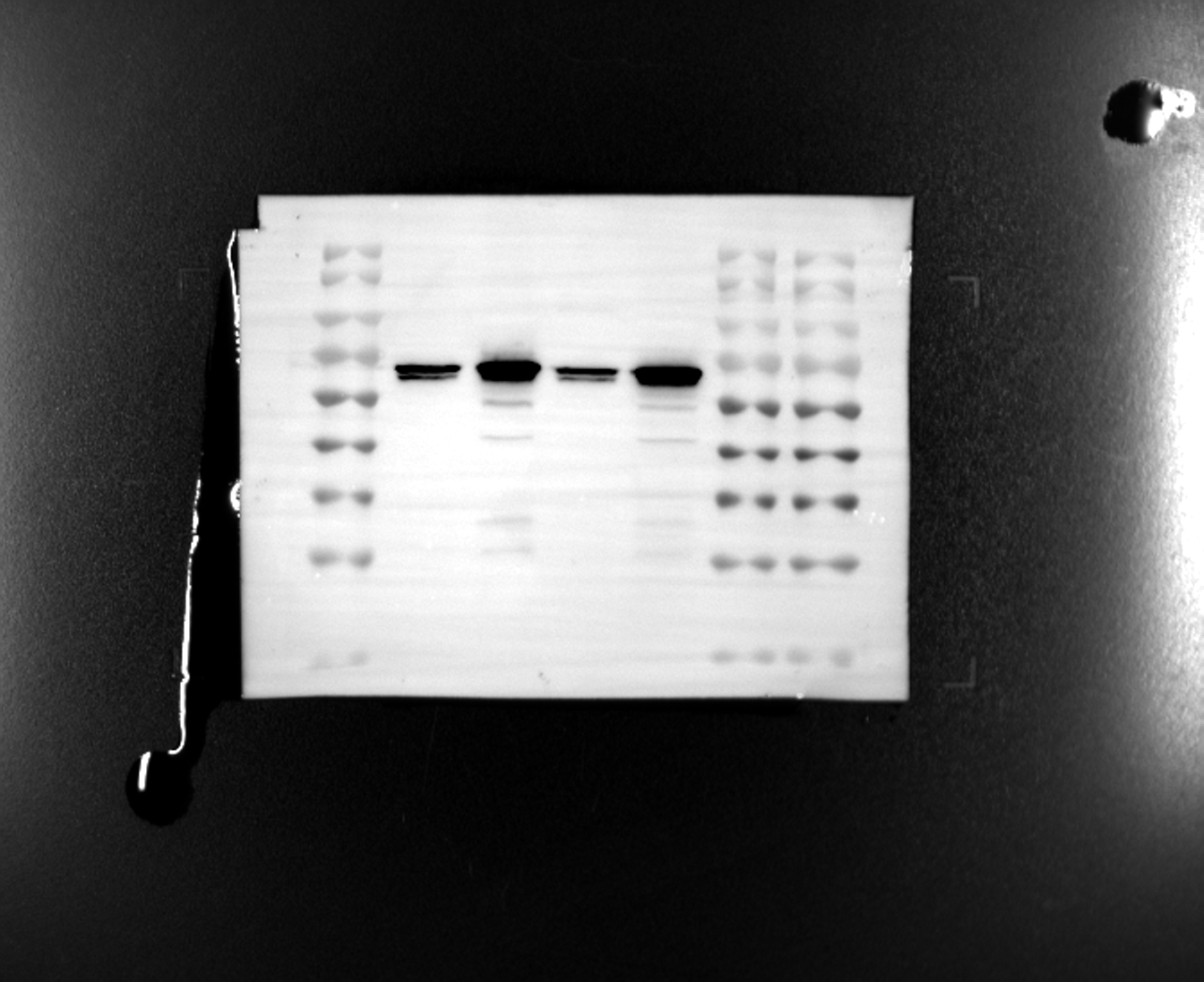

Supplement: Supplementary file 1 — Supplementary Table 1 Primer sequences of STARD12/14 and GAPDH; Supplementary Table 2 The significant differences of transcriptional expression of STARDs between diverse types of LUAD and normal samples (Oncomine); Supplementary Table 3 Univariate and multivariate Cox analysis of STARD12 and STARD14 in LUAD patients; Supplementary Table 4 Enrichment analysis results of STARDs in LUAD; Supplementary Figure 1 Pathological histological subgroup expression analysis of STARDs in LUAD; Supplementary Figure 2 Survival analysis on DFS of STARDs in LUAD; Supplementary Figure 3 Survival analysis on PFS of STARDs in LUAD; Supplementary Figure 4 Survival analysis on DSS of STARDs in LUAD. [file ijmsv20p1427s1.zip › Supplementary materials/Western blot-Raw data/whole gel/STARD14/STARD14-R.Tif]

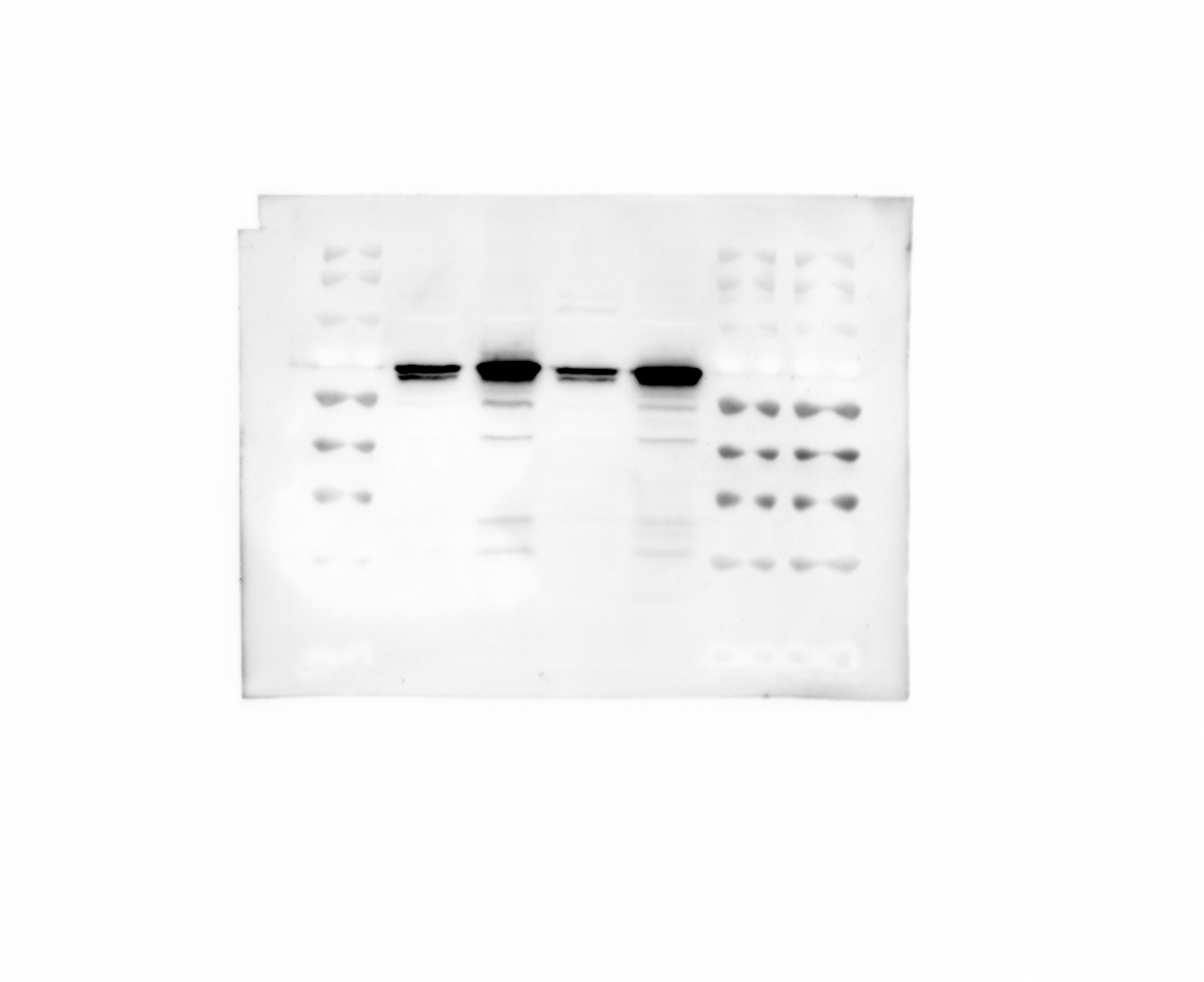

Supplement: Supplementary file 1 — Supplementary Table 1 Primer sequences of STARD12/14 and GAPDH; Supplementary Table 2 The significant differences of transcriptional expression of STARDs between diverse types of LUAD and normal samples (Oncomine); Supplementary Table 3 Univariate and multivariate Cox analysis of STARD12 and STARD14 in LUAD patients; Supplementary Table 4 Enrichment analysis results of STARDs in LUAD; Supplementary Figure 1 Pathological histological subgroup expression analysis of STARDs in LUAD; Supplementary Figure 2 Survival analysis on DFS of STARDs in LUAD; Supplementary Figure 3 Survival analysis on PFS of STARDs in LUAD; Supplementary Figure 4 Survival analysis on DSS of STARDs in LUAD. [file ijmsv20p1427s1.zip › Supplementary materials/Western blot-Raw data/whole gel/STARD14/STARD14.Tif]
